# In Situ Programming of the Tumor Microenvironment to Alleviate Immunosuppression for Pancreatic Cancer Immunotherapy

**DOI:** 10.1002/advs.202504008

**Published:** 2025-06-23

**Authors:** Man Sun, Huan Zhang, Yarui Ma, Simiao Wang, Jiayi Chen, Yaxin Cui, Yun Zhang, Siyuan Hu, Dan Zhou, Pengchen Zhang, Yahui Liu, Betty Y.S. Kim, Wen Jiang, Xiaobing Wang, Zhaogang Yang

**Affiliations:** ^1^ School of Life Sciences Jilin University Changchun 130012 China; ^2^ State Key Laboratory of Molecular Oncology National Cancer Center/National Clinical Research Center for Cancer/Cancer Hospital Chinese Academy of Medical Sciences and Peking Union Medical College Beijing 100021 China; ^3^ Department of Hepatobiliary and Pancreatic Surgery The First Hospital of Jilin University Changchun 130021 China; ^4^ Department of Neurosurgery The University of Texas MD Anderson Cancer Center Houston TX 77030 USA; ^5^ Department of Radiation Oncology The University of Texas MD Anderson Cancer Center Houston TX 77030 USA

**Keywords:** cancer immunotherapy, cGAS‐STING, drug delivery, exosomes, pancreatic cancer

## Abstract

Recent studies have highlighted the pivotal role of the cGAS‐STING pathway in cancer immunotherapy. However, clinical trials with cGAS‐STING pathway agonists have faced setbacks thanks to their short biological half‐life, lack of tumor specificity, and potential to promote tumor immune evasion. To address these challenges, a novel exosome‐based drug delivery platform, termed cmExo^aCD11b^ is developed, designed to precisely target and reprogram the tumor microenvironment (TME) in situ for pancreatic cancer immunotherapy. cmExo^aCD11b^ is engineered to encapsulate high copy numbers of IL‐12 mRNA and 2′3’‐cGAMP (cGAMP) and is functionalized with CD11b antibodies for targeted delivery to macrophages. Notably, cmExo^aCD11b^ facilitated the repolarization of M2 macrophages to M1 phenotype, thereby reprogramming the TME and enhancing the secretion of pro‐inflammatory cytokines. This immunomodulatory effect reversed the immunosuppressive milieu of the TME and significantly inhibited tumor progression. More importantly, cmExo^aCD11b^ exhibited robust therapeutic efficacy in both murine pancreatic cancer and patient‐derived xenograft models. These results suggest that cmExo^aCD11b^ represents a promising approach for overcoming immunosuppression in pancreatic cancer, paving the way for its potential application in cancer immunotherapy.

## Graphical abstract

cmExo^aCD11b^ induced the repolarization of immunosuppressive M2 macrophages into pro‐inflammatory M1 phenotype, thereby promoting the proliferation and activation of CD8^+^ T cells and reversing the immunosuppressive state of the tumor microenvironment.

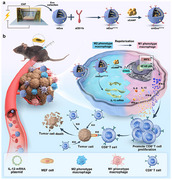

## 1 Introduction

Over the past few years, STING (stimulator of interferon genes) receptors have emerged as pivotal activators of both innate and adaptive immune responses against cancer through the activation of the interferon (IFN) signaling pathway.^[^ ^1^ ^]^ A common therapeutic strategy for leveraging the STING pathway involves the direct delivery of agonists to tumor sites. However, this approach is hindered by the tumor's capacity to induce STING‐mediated T cell death.^[^ ^2^ ^]^ Moreover, clinical trials utilizing intratumoral injection of STING agonists have been discontinued due to their limited efficacy.^[^ ^3^, ^4^ ^]^ Although some studies have attempted to address these challenges by delivering agents to antigen‐presenting cells (APCs), this method has inadvertently resulted in the enhanced expression of PD‐L1 on cancer cells, promoting immune escape.^[^ ^5^, ^6^, ^7^ ^]^ To counteract this, researchers have explored combining STING pathway activation with immune checkpoint blockade to amplify the anti‐cancer immunity. However, immune checkpoint blockade has demonstrated sustained efficacy in only a limited subset of cancer patients, with many experiencing tumor recurrence after initial responses.^[^ ^8^, ^9^ ^]^ Furthermore, the efficacy of PD‐L1 antibodies is significantly limited by their rapid degradation and clearance via the reticuloendothelial system (RES) following systemic administration.^[^ ^10^, ^11^ ^]^ This underscores the urgent need for an intelligent delivery platform capable of effectively activating the STING pathway while circumventing tumor immunosuppression. Such a platform would also need to elicit a sustained immunotherapeutic response, ultimately improving the overall efficacy of cancer immunotherapy.

**Figure 1:**
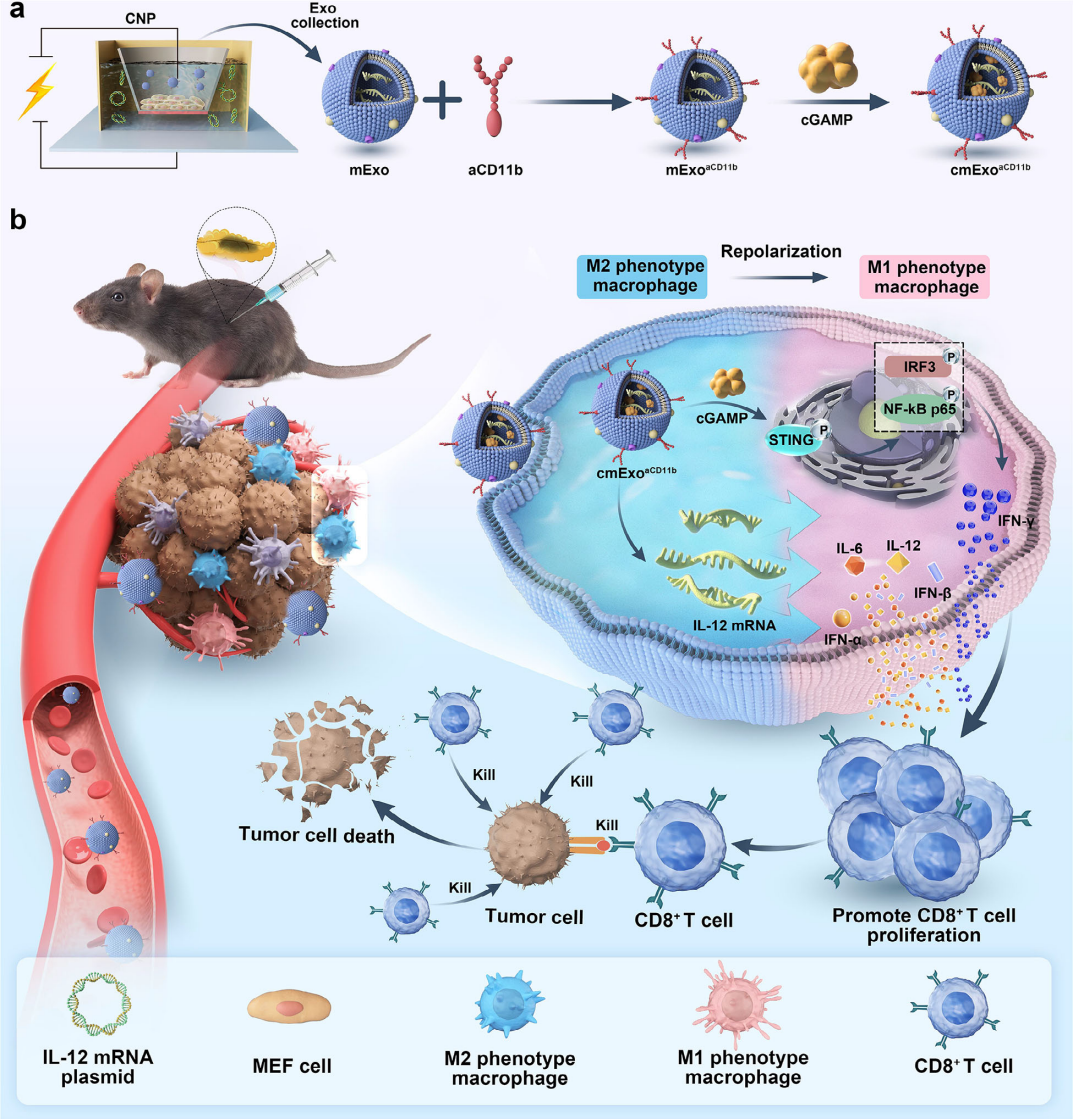
Schematic representation of the preparation and immune activation process of cmExo^aCD11b^ for pancreatic cancer immunotherapy. a) Construction of cmExo^aCD11b^: IL‐12 mRNA plasmids were transiently transfected into mouse embryonic fibroblasts (MEF) via CNP technology, facilitating the endogenous loading of IL‐12 mRNA into Exo. Subsequently, anti‐CD11b antibodies (aCD11b) were coupled onto the Exo surface via lipid embedding, and 2′3’‐cGAMP (cGAMP) was loaded into Exo using a sonication method. b) Therapeutic mechanism of cmExo^aCD11b^: Upon delivery to the TME, cGAMP activates the cGAS‐STING pathway, enhancing IFN production and promoting T cell activation. Concurrently, IL‐12 promotes the repolarization of M2 macrophages into M1 phenotype, effectively reversing the immunosuppressive tumor microenvironment (iTME) and promoting CD8^+^ T cell proliferation, and ultimately suppressing tumor growth. Abbreviations: mExo: Exo containing IL‐12 mRNA; mExo^aCD11b^: mExo modified with aCD11b; cmExo^aCD11b^: mExo^aCD11b^ loaded with cGAMP.

Interleukin‐12 (IL‐12) is a pro‐inflammatory cytokine with potent tumor‐suppressive properties. It plays a pivotal role in modulating the tumor microenvironment (TME) by promoting macrophage polarization toward the M1 phenotype, thereby inducing the secretion of additional pro‐inflammatory cytokines and enhancing antitumor immune responses.^[^ ^12^ ^]^ Upon activation, M1 macrophages not only facilitate the infiltration of CD8^+^ T cells but also suppress the expansion of immunosuppressive regulatory T cells (Tregs), thereby serving as critical mediators in reversing immunosuppression and bolstering a robust innate immune response.^[^ ^13^ ^]^ Based on these immunomodulatory properties, we sought to further reprogram the TME by incorporating IL‐12 mRNA into our therapeutic strategy. Exosome (Exo), characterized by their superior biocompatibility, biosafety, notable stability, and ability to penetrate physiological barriers, has emerged as promising drug carriers.^[^ ^14^, ^15^, ^16^, ^17^ ^]^ However, their potential as a platform for selectively activating the STING pathway and alleviating tumor immunosuppression remains underexplored. To address this problem, we engineered a novel Exo‐based delivery system, termed cmExo^aCD11b^, designed to activate the STING signaling, reprogram tumor‐associated macrophages (TAMs), reducing the tumor immunosuppression in TME. Specifically, cmExo^aCD11b^ was engineered using cellular nanoporation (CNP) technology to efficiently encapsulate high copy numbers of IL‐12 mRNA, thereby enhancing its therapeutic efficacy.^[^ ^18^ ^]^ 2′3’‐cGAMP (cGAMP) was then co‐encapsulated within Exo to ensure robust activation of the STING pathway. Meanwhile, cmExo^aCD11b^ activated the STING pathway by specifically targeting macrophages, facilitated by the modification of anti‐CD11b antibodies (aCD11b) on the surface of Exo, leading to stable and sustained expression of IFN. Upon reaching the TME, cmExo^aCD11b^ induced the repolarization of immunosuppressive M2 macrophages into pro‐inflammatory M1 phenotype, thereby promoting the proliferation and activation of CD8^+^ T cells and reversing the immunosuppressive state of the TME (**Figure** **1**). This innovative Exo‐based delivery platform offers a promising strategy for achieving stable STING pathway activation and precise immune modulation within the TME using a single integrated system, ultimately improving the efficacy and safety of pancreatic cancer immunotherapy.

## 2 Results and Discussion

### 2.1 Preparation and Characterization of cmExo^aCD11b^

**Figure 2:**
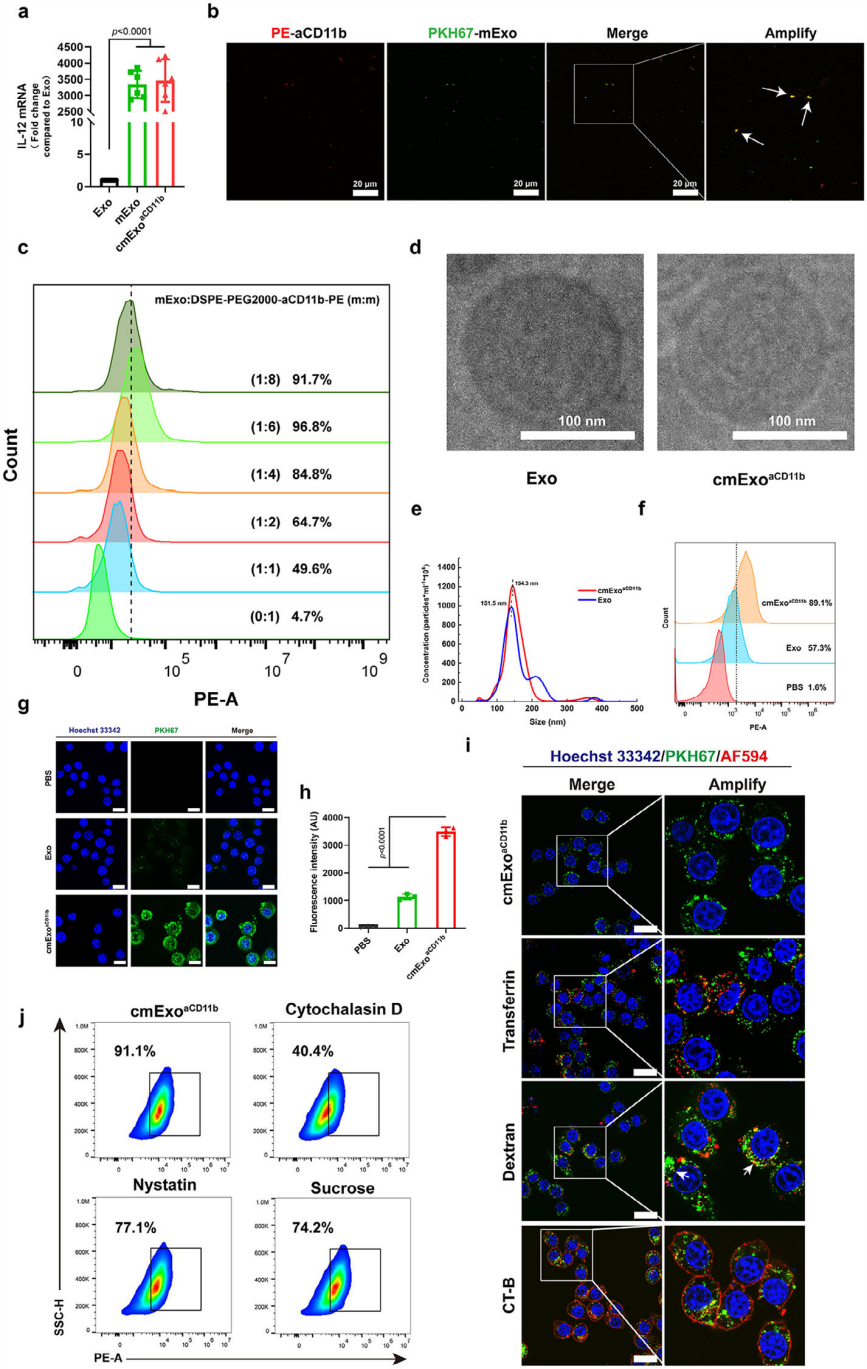
Construction and characterization of cmExo^aCD11b^. a) Loading capacity of IL‐12 mRNA into cmExo^aCD11b^, analyzed by RT‐qPCR. b) Representative confocal laser scanning microscopy images showing the successful coupling of mExo with aCD11b. Arrow: mExo with aCD11b adsorbed. Scale bar: 20 µm. c) Binding affinity of mExo to aCD11b‐PE, measured by flow cytometry. d) Cryo‐TEM images showing the double‐layered vesicle structure both cmExo^aCD11b^ and Exo. Scale bar: 100 nm. e) Particle size distributions of cmExo^aCD11b^ and Exo determined by NTA. f) Flow cytometric quantification of PKH26‐cmExo^aCD11b^ uptake in RAW264.7 cells after 4 h incubation. g) Cellular uptake of PKH67‐cmExo^aCD11b^ by RAW264.7 cells, determined by confocal laser scanning microscopy. Scale bar: 20 µm. h) Average fluorescence intensity of cellular uptake, detected by confocal laser scanning microscopy. i) Cellular uptake mechanisms of cmExo^aCD11b^ by RAW264.7 cells, determined by confocal laser scanning microscopy. Arrow: cmExo^aCD11b^ co‐localized with Dextran. Scale bar: 20 µm. CT‐B: Cholera toxin subunit B conjugates. j) Results of inhibitor experiments quantified by flow cytometry demonstrated that RAW264.7 cells internalize cmExo^aCD11b^ via macropinocytosis‐mediated endocytosis. SSC‐H: Side Scatter Height. Data are presented as mean ± s.d.; statistical significance was calculated by one‐way ANOVA followed by Tukey’s multiple comparisons test.

In this work, we developed a novel Exo‐based drug delivery system co‐loaded with IL‐12 mRNA and cGAMP using CNP technology and sonication method. This system is designed to active the cGAS‐STING pathway and promote the polarization of M2 macrophages into M1 phenotype, aiming to achieve efficient immunotherapy for pancreatic cancer. First, we utilized CNP technology to generate Exo containing IL‐12 mRNA (mExo), achieving a ≈ 3300‐fold increase in IL‐12 mRNA loading within Exo compared to the control group (**Figure** **2**a). To specifically target macrophages in the TME, mExo was further modified with aCD11b, leveraging the high expression of CD11b on macrophage surfaces. The successful attachment of aCD11b to the mExo was confirmed using laser confocal microscopy (Figure 2b). Additionally, the optimal conjugation ratio of mExo to aCD11b was determined to be 1:6 (mExo:DSPE‐PEG2000‐aCD11b‐PE, m:m) (Figure 2c), ensuring efficient targeting and functionality. Next, cGAMP through sonication was further loaded into the mExo^aCD11b^ obtained in the previous step, and the final product, cmExo^aCD11b^, was obtained. Additionally, the loading efficiency of cGAMP in cmExo^aCD11b^ was assessed, achieving a drug loading efficiency of 3.06 ± 0.04% when the mass ratio of mExo^aCD11b^ and cGAMP was 5:1 (Table S1, Supporting Information). Furthermore, signature exosomal proteins, such as CD9 and CD63, were significantly enriched in both the Exo and cmExo^aCD11b^ groups, indicating successful preparation of cmExo^aCD11b^ (Figure S1, Supporting Information). Moreover, cryogenic transmission electron microscopy (cryo‐TEM) analysis revealed a typical circular bilayer vesicle structure with uniform size distribution in the cmExo^aCD11b^ group (Figure 2d; Figure S2, Supporting Information). The particle size of cmExo^aCD11b^, measured by Nanoparticle Tracking Analyzer (NTA), showed an average diameter of ≈150 nm (Figure 2e). After the modification of aCD11b on the surface of cmExo^aCD11b^, its ζ‐potential changed from −12.4 to −10.2 mV, as compared to Exo (Figure S3, Supporting Information). Furthermore, cmExo^aCD11b^ exhibited excellent stability, maintaining a consistent particle size over a 7‐day period and preserving the integrity of encapsulated IL‐12 mRNA in whole blood within 24 h (Figure S4a,b, Supporting Information). The uptake of cmExo^aCD11b^ in RAW264.7 cells was higher than that of Exo, attributed to the coupling of aCD11b (Figure 2f–h). Similar results were also observed in THP‐1 cells (Figure S5a–c, Supporting Information). In contrast, in Panc02 cells, which are CD11b negative, the overall uptake was lower than that in RAW264.7 and THP‐1 cells, with no difference in uptake between the cmExo^aCD11b^ and Exo groups (Figure S5c,d, Supporting Information). These results demonstrated that cmExo^aCD11b^ exhibits a strong targeting ability toward macrophages. In addition, the cellular uptake mechanism of cmExo^aCD11b^ was also investigated. The results displayed high co‐localization of cmExo^aCD11b^ with dextran, as along with partial co‐localization with other endocytosis biomarkers, suggesting that the cmExo^aCD11b^ primarily enters macrophages through macropinocytosis‐mediated endocytosis (Figure 2i; Figure S6, Supporting Information). Interestingly, this phenomenon was inhibited by Cytochalasin D, an endocytosis blocker, further confirming the importance of macropinocytosis‐mediated endocytosis in the cellular uptake of cmExo^aCD11b^ (Figure 2j). Additionally, IL‐12 protein expression was successfully detected 24 h after cmExo^aCD11b^ treatment in both RAW264.7 cells and THP‐1 cells, thereby establishing a foundation for subsequent IL‐12‐mediated immunotherapy (Figure S7, Supporting Information). Based on these findings, it was demonstrated that cmExo^aCD11b^, encapsulating high copy numbers of IL‐12 mRNA and cGAMP, was successfully prepared and holds potential for pancreatic cancer immunotherapy through macrophage targeting.

### 2.2 The Immune Activation by cmExo^aCD11b^ In Vitro

**Figure 3:**
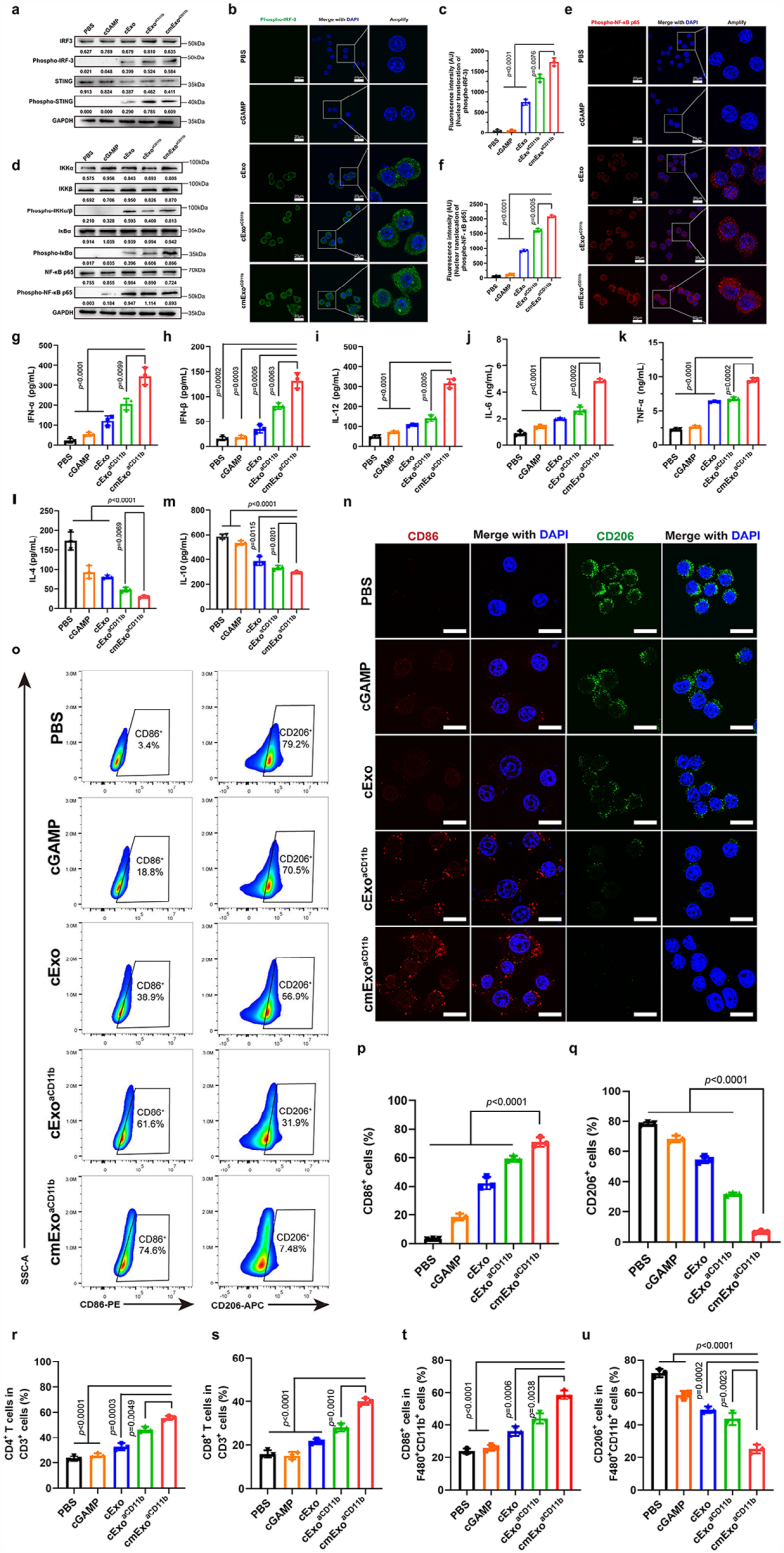
Activation of the STING pathway by cmExo^aCD11b^. a) Western blot analysis of STING‐IRF3 pathway proteins in RAW264.7 cells treated with cGAMP, cExo, cExo^aCD11b^, or cmExo^aCD11b^ for 6 h. Both cExo^aCD11b^ and cmExo^aCD11b^ strongly activated the STING‐IRF3 pathway. Band intensities were quantified using ImageJ software, and the expression of each protein was normalized to the corresponding GAPDH signal. b) Confocal laser scanning microscopy images showing nuclear translocation of activated IRF3 (phospho‐IRF‐3, green) in RAW264.7 cells (blue) after treatment with cmExo^aCD11b^ for 6 h. Both cExo^aCD11b^ and cmExo^aCD11b^ induced nuclear translocation, with cmExo^aCD11b^ exhibiting a stronger nuclear translocation effect. Scale bar: 20 µm. c) Average fluorescence intensity of phospho‐IRF‐3 nuclear translocations in each group. d) Western blot analysis of activated NF‐κB p65 in RAW264.7 cells treated with cGAMP, cExo, cExo^aCD11b^, or cmExo^aCD11b^ for 6 h. Both cExo^aCD11b^ and cmExo^aCD11b^ strongly activated the NF‐κB pathway. Band intensities were quantified using ImageJ software, and the expression of each protein was normalized to the corresponding GAPDH signal. e) Confocal laser scanning microscopy images of the nuclear translocation of activated phospho‐NF‐κB p65 in RWA264.7 cells. Scale bar: 20 µm. f) Average fluorescence intensity of phospho‐NF‐κB p65 nuclear translocations in each group. g,h) After 6 h of co‐incubation of RAW264.7 cells with drugs from each experimental group, the levels of IFN‐α and IFN‐β in the cell culture supernatants were analyzed using ELISA (*n* = 3). i–m) Following a 48 h co‐incubation of RAW264.7 cells with drugs from each experimental group, cytokine secretion levels in the cell culture supernatants were quantified via ELISA (*n* = 3). n) The level of CD86 and CD206 in RAW264.7 cells, as determined by confocal laser scanning microscopy. Scale bar: 10 µm. o) Flow cytometry analysis of CD86 and CD206 level in RAW264.7 cells (*n* = 3). SSC‐A: Side Scatter Area. p,q) Proliferation ratio of CD86^+^ and CD206^+^ cell in each group. r,s) Proliferation of CD4^+^ and CD8^+^ T cells in CD3^+^ cells measured by flow cytometry after activation of bone marrow‐derived macrophages (BMDMs) by cmExo^aCD11b^ and OVA peptides for 72 h (*n* = 3). t,u) Proliferation rate of CD86^+^ and CD206^+^ cells in F480^+^CD11b^+^ cells measured by flow cytometry after activation of BMDMs with cmExo^aCD11b^ and OVA peptides for 72 h (*n* = 3). Data are presented as mean ± s.d.; statistical significance was calculated by one‐way ANOVA followed by Tukey’s multiple comparisons test (c, f, g‐m, p‐u).

To validate that STING can be activated by cmExo^aCD11b^, we examined the phosphorylation status of STING and IFN regulatory factor 3 (IRF‐3). As expected, strong phosphorylation of STING and IRF‐3 was observed in both RAW264.7 and THP‐1 cells after 6 h of treatment with cmExo^aCD11b^. In contrast, no significant phosphorylation was detected in cells treated with cGAMP alone, likely due to its limited cellular uptake (**Figure** **3**a; Figure S8, Supporting Information). Furthermore, to confirm the activation of the STING signaling, we investigate the nuclear translocation of phospho‐IRF‐3 in both RAW264.7 and THP‐1 cells using confocal laser scanning microscopy. Nuclear translocation of phospho‐IRF‐3 was clearly observed in macrophages after 6 h of treatment with cmExo^aCD11b^, suggesting that phospho‐IRF‐3 was activated as a transcription factor for pro‐inflammatory genes (Figure 3b,c; Figure S9, Supporting Information). Similarly, phospho‐NF‐κB p65, another key downstream component of the cGAS‐STING signaling, was also activated following this treatment (Figure 3d; Figure S10, Supporting Information). Additionally, the nuclear translocation of phospho‐NF‐κB p65 in macrophages further confirmed the activation of NF‐κB (Figure 3e,f; Figure S11, Supporting Information). Since the activation of both IRF‐3 and NF‐κB signaling pathway triggers the production of IFN, ultimately leading to T cell activation, we investigated the expression of IFN in the supernatant of both RAW264.7 and THP‐1 cells following cmExo^aCD11b^ treatment. The results showed that the STING activation induced a type I IFN pro‐inflammatory pathway, with increased expression of IFN‐α and IFN‐β (Figure 3g,h; Figure S12, Supporting Information).

To evaluate the potential of cmExo^aCD11b^ in regulating macrophage repolarization, RAW264.7 cells were first stimulated and polarized into M2 phenotype macrophages using IL‐4 and IL‐13 cytokine in vitro as described (Figure S13, Supporting Information).^[^ ^19^ ^]^ After treating M2 phenotype RAW264.7 cells with cmExo^aCD11b^ for 48 h, the secretion of pro‐inflammatory cytokines (IL‐12, IL‐6, and TNF‐α), was significantly enhanced. In contrast, the releases of anti‐inflammatory cytokines, such as IL‐4 and IL‐10, were downregulated, suggesting the successful repolarization of M2 macrophages into the M1 phenotype (Figure 3i–m). Moreover, these findings were also observed in THP‐1 cells (Figure S14, Supporting Information). To further confirm macrophage repolarization, the expression of M1 (CD86) and M2 (CD206) phenotype biomarkers was measured using confocal laser scanning microscopy. Compared to other groups, the cmExo^aCD11b^ treated group showed significantly higher CD86 expression and significantly lower CD206 expression (Figure 3n; Figure S15, Supporting Information). Furthermore, flow cytometry results corroborated that M2 phenotype macrophages were repolarized into M1 phenotype (Figure 3o–q; Figure S16, Supporting Information), consistent with the findings above. Additionally, to further investigate the effects of cmExo^aCD11b^, we simulated the TME in vitro. The number of CD4^+^ and CD8^+^ T cells was dramatically increased in the cmExo^aCD11b^ group (Figure 3r,s). There was also an increase in the proportion of M1 phenotype and a dramatical decrease in the proportion of M2 phenotype (Figure 3t,u). These results suggest that cmExo^aCD11b^ effectively enhances STING activation within the TME, promotes macrophage repolarization, and facilitates T cell activation.

### 2.3 The Systemic Antitumor Response by cmExo^aCD11b^

To assess the therapeutic potential of cmExo^aCD11b^ for cancer immunotherapy in vivo, its biosafety was first assessed using a hemolysis assay. Erythrocyte precipitation was observed, but no obviously hemolysis was detected in the cmExo^aCD11b^ group across various concentrations tested (Figure S17, Supporting Information). Furthermore, biochemical markers of liver and kidney function, including alanine aminotransferase (ALT), aspartate aminotransferase (AST), creatinine, and blood urea nitrogen (BUN), showed no significant difference between cmExo^aCD11b^‐treated and PBS‐treated mice 24 h post‐ administration, indicating excellent biosafety (Figure S18, Supporting Information). Collectively, these results demonstrate that cmExo^aCD11b^ exhibits excellent biosafety for in vivo administration.

**Figure 4:**
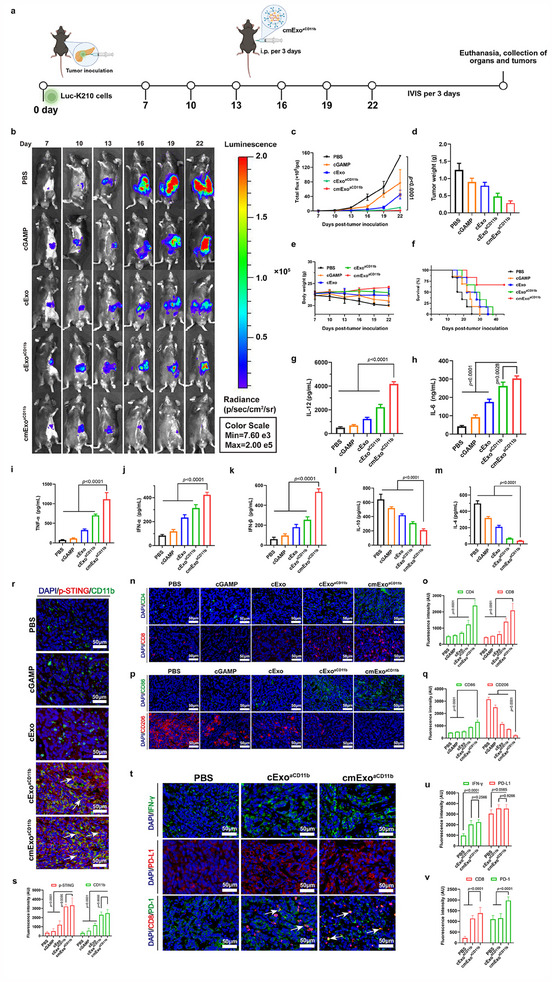
In vivo evaluation of cmExo^aCD11b^ for pancreatic cancer immunotherapy. a) Schematic representation of the cancer therapeutic process. Mice were subjected to IVIS monitoring and intraperitoneal injection at 3‐day intervals (*n* = 6). b,c) Representative IVIS images and quantified signal intensity of Luc‐K210 cells pancreatic tumors over 22 days. d) Tumor weight of mice in each group. e) Body weight changes of mice monitored over a 22‐day period. f) Survival rates of mice in each experimental group monitored over 45 days, with survival curves generated accordingly. g–m) Serum levels of cytokines IL‐12, IL‐6, TNF‐α, IFN‐α, IFN‐β, IL‐10, and IL‐4 evaluated by ELISA (*n* = 3). n) Immunofluorescence staining of CD4^+^ and CD8^+^ T cells in tumor tissues. Scale bar: 50 µm. o) Quantification of average fluorescence intensity for CD4 and CD8 T cells. p) Immunofluorescence staining of CD86 and CD206 in tumor tissues. Scale bar: 50 µm. q) Quantification of average fluorescence intensity for CD86 and CD206 cells. r) Immunofluorescence staining of p‐STING in CD11b^+^ cells within tumor tissues. Arrow: p‐STING co‐localized with CD11b. Scale bar: 50 µm. s) Quantification of average fluorescence intensity for p‐STING and CD11b protein. t) Immunofluorescence staining of IFN‐γ and PD‐L1 in tumor tissues. PD‐1 expression in CD8^+^ T cells within tumor samples was investigated. Arrow: PD‐1 co‐localized with CD8. Scale bar: 50 µm. u,v) Quantification of average fluorescence intensity for IFN‐γ, PD‐L1, CD8, and PD‐1 expression. All data are presented as mean ± s.d.; statistical significance was calculated by one‐way ANOVA followed by Tukey’s multiple comparisons test (c‐m, o, q, s, u, v).

To assess the therapeutic efficacy of cmExo^aCD11b^ for pancreatic cancer in vivo, an orthotopic pancreatic cancer mouse model was first established. cmExo^aCD11b^ was administered intraperitoneally every 3 days from day 7 post‐modeling, as illustrated in **Figure** **4**a. Compared to other treatment groups, cmExo^aCD11b^ administration resulted in a remarkable inhibition of tumor growth, as evidenced by a substantial reduction in tumor volume and weight 22 days after tumor implantation, with minimum effects on the body weight of mice (Figure 4b–e). Meanwhile, the median survival time was dramatically extended from 16 days in the control group to over 45 days in the cmExo^aCD11b^ group (Figure 4f). Immunohistochemical (IHC) staining of tumor sections further confirmed the inhibitory effect of cmExo^aCD11b^ on tumor cell proliferation, as evidenced by the inhibited expression of Ki67 in the cmExo^aCD11b^ group. Notably, minimal inhibitory effects on tumor cell proliferation were observed in other groups (Figure S19, Supporting Information). In addition, H&E staining of organs from each treatment group revealed no signs of tissue damage (Figure S20, Supporting Information). Additionally, pro‐inflammatory cytokines, such as IL‐12, IL‐6, TNF‐α, IFN‐α, and IFN‐β, were dramatically increased. In contrast, anti‐inflammatory cytokines, including IL‐10 and IL‐4, were reduced in the peripheral blood of mice treated with cmExo^aCD11b^ in 24 h after drug administration (Figure 4g–m). To further assess the immune response within the tumor tissue, immunofluorescence staining of the tumor tissue was conducted. The results showed an enhanced presence of both CD4^+^ and CD8^+^ T cells in cmExo^aCD11b^ group compared to other groups, aligning with our expectations (Figure 4n,o). Moreover, the number of CD86^+^ cells, representing M1 macrophages, was marked increased in the tumor tissue of the cmExo^aCD11b^ group (Figure 4p,q; Figure S21, Supporting Information). In contrast, the number of CD206^+^ cells, representing M2 macrophages, was decreased. These findings suggest that cmExo^aCD11b^ treatment effectively induced the repolarization of M2 macrophages into M1 macrophages within the TME.

Next, to elucidate the molecular mechanism underlying cmExo^aCD11b^‐mediated tumor eradication, we performed immunofluorescence analysis of tumor tissues 22 days post‐administration. The results showed that cmExo^aCD11b^ effectively activated the STING pathway in vivo. Moreover, co‐localization analysis of CD11b with phospho‐STING (p‐STING) demonstrated that STING pathway activation predominantly occurs in CD11b‐positive cells (Figure 4r,s). Various reports have confirmed that activation of the STING signaling stimulates the release of IFN‐γ from APCs, which subsequently induces PD‐L1 expression in tumor cells, facilitating tumor immune escape from T cell eradication.^[^ ^20^, ^21^, ^22^ ^]^ Consistent with these findings, our results showed a significant enhancement in IFN‐γ expression in the cExo^aCD11b^ group (Figure 4t). Correspondingly, PD‐L1 expression was notably increased in the tumor tissue following cExo^aCD11b^ treatment (Figure 4t–v). A similar trend in both IFN‐γ and PD‐L1 expression was recorded in the cmExo^aCD11b^ group, compared to cExo^aCD11b^ treatment. Moreover, in the cmExo^aCD11b^ group, an enhanced expression of PD‐1 on CD8^+^ T cells was observed within the TME. Based on these findings, cmExo^aCD11b^ exhibits significant potential for tumor elimination by facilitating the repolarization of M2 macrophages into M1 phenotype within the TME.

### 2.4 cmExo^aCD11b^ Causes Reorganization of Macrophages in the TME

**Figure 5:**
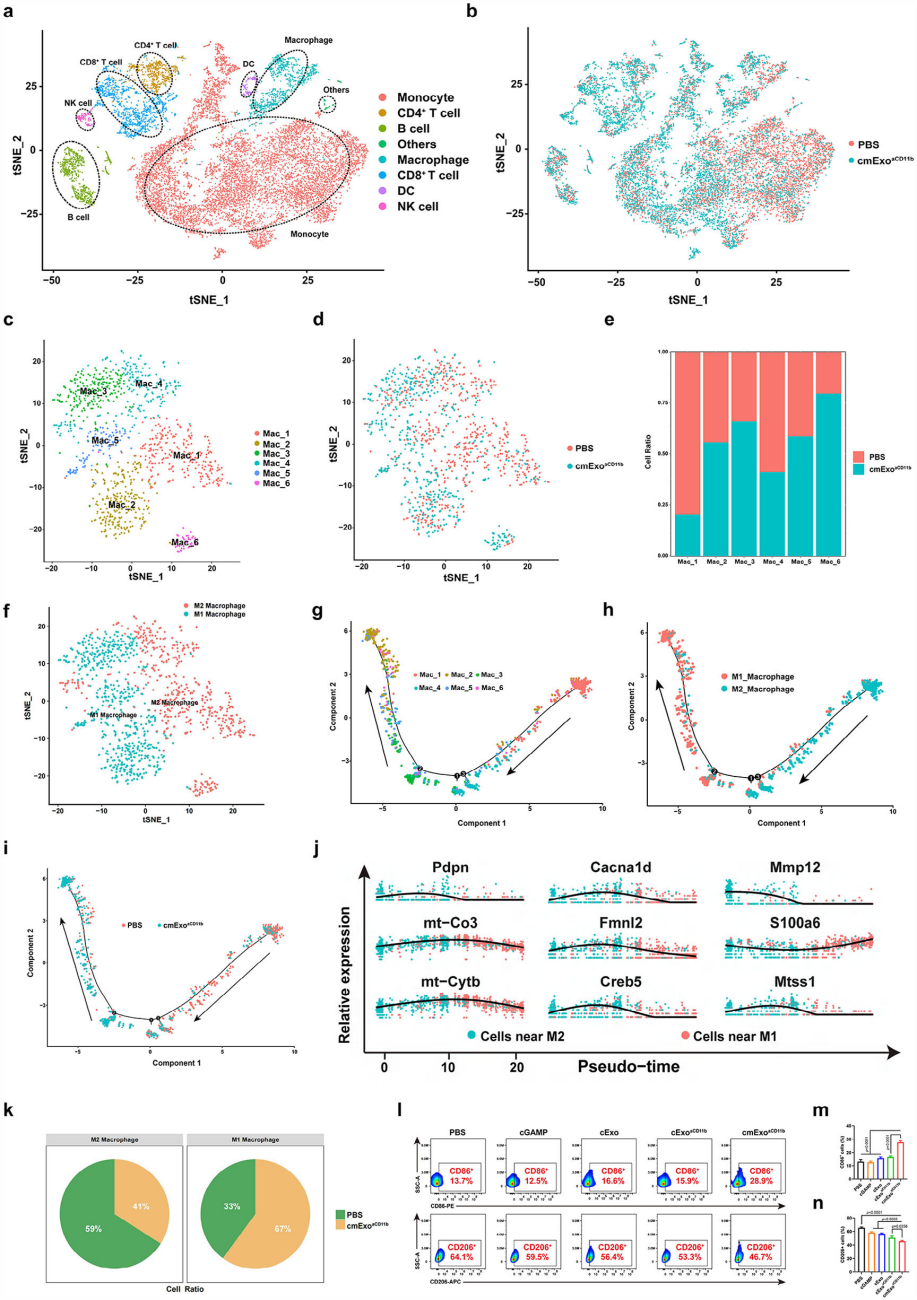
cmExo^aCD11b^ reprogram the macrophage phenotype in the TME. a) Unsupervised scRNA‐seq of pooled immune cells (*n* = 3 mice per group) obtained 48 h post‐treatment revealed 8 color‐coded cell clusters. b) t‐SNE visualization of all cells stratified by treatment group. c) t‐SNE subclustering of 6 color‐coded macrophage subsets combined from both treatment groups. d,e) t‐SNE plot, color‐coded by treatment (c) and bar plot (e) showing subpopulations of macrophages distinctly separated between PBS and cmExo^aCD11b^ treatment groups. f) t‐SNE plot illustrating the spatial distribution of M1 and M2 phenotype macrophage. g) Superimposition of macrophage subclusters (Mac_1 to Mac_6) on a pseudotime trajectory. h) Superimposition of macrophage subclusters (M1, M2) on a pseudotime trajectory. i) Superimposition of macrophage subclusters (PBS, cmExo^aCD11b^) on a pseudotime trajectory. j) Scatter plot showing the gene expression levels of transcription factors associated with M2 to M1 repolarization as a function of pseudotime, with each dot representing the expression of a single cell. k) scRNA‐seq‐based visualization illustrating the changes in M1 and M2 macrophage populations under different treatment groups. l) Flow cytometry analysis showing a significantly increase in the expression of CD86 protein, indicative of enhanced M1 phenotype macrophages following cmExo^aCD11b^ treatment. m,n) Quantitative analysis of the proportion of CD86^+^ cells and CD206^+^ cells within mouse tumor tissue. All data are presented as mean ± s.d. (*n* = 3); Statistical significance was calculated by one‐way ANOVA followed by Tukey’s multiple comparisons test.

To investigate the immune landscape modulated by the cmExo^aCD11b^ platform and its influence on the TME, single‐cell RNA sequencing (scRNA‐seq) technology was employed to delineate cell‐type‐specific transcriptional profiles within the TME of mice subjected to different treatments. Approximately 7800 individual immune cells derived from pancreatic tumors, with 3 mice per treatment condition, were analyzed using scRNA‐seq. The t‐distributed stochastic neighbor embedding (t‐SNE) projection showed 8 distinct cell clusters identified through unsupervised clustering. These clusters included various immune cell types, including monocytes, macrophages, CD4^+^ T cells, CD8^+^ T cells, dendritic cells (DCs), NK cells, and B cells (**Figure** **5**a). Applying the aforementioned immune cells to the treatment groups, it is observed that almost all of them presented a significant enhance in number following cmExo^aCD11b^ induction (Figure 5b). Next, focusing on macrophages, they were further categorized into 6 distinct subclusters, allowing for a detailed examination of their phenotypic changes (Figure 5c). Following cmExo^aCD11b^ treatment, clusters Mac_2, Mac_3, Mac_5, and Mac_6 were predominantly enriched and significantly upregulated (Figure 5d,e). These clusters were further characterized based on marker genes associated with M1 and M2 phenotype macrophages (Figure 5f). Specifically, Mac_1, Mac_4, and Mac_6 were predominantly defined as M2 phenotype macrophages, whereas Mac_2, Mac_3, and Mac_5 were identified as M1 phenotype macrophages. Notably, Mac_1 and Mac_4 appeared to play a significant role in cmExo^aCD11b^‐induced M1 macrophage infiltration. Next, to explore the transcriptional dynamics underlying macrophage repolarization, we employed the Monocle2 trajectory analysis method. This approach revealed two distinct cell fates—M2 and M1—representing alternative polarization states (Figure 5g; Figure S22, Supporting Information). Consistent with their phenotypic classification, Mac_1, Mac_4, and Mac_6 were localized closer to the M2 phenotype, whereas Mac_2, Mac_3, and Mac_5 were positioned closer to the M1 phenotype (Figure 5g,h). Notably, cmExo^aCD11b^ treatment induced a pronounced macrophage repolarization, driving a progressive shift from an M2‐dominated phenotype toward an M1‐dominated phenotype (Figure 5i). During this transition, genes exhibiting progressively increasing or decreasing expression patterns were observed, highlighting the dynamic regulation of macrophage polarization (Figure 5j). Furthermore, Gene Ontology (GO) enrichment and Kyoto Encyclopedia of Genes and Genomes (KEGG) pathway analyses were performed to delineate the biological processes involved in macrophage polarization. The results revealed a significant activation of immune‐related pathways during the transition from the M2 to the M1 phenotype, along with marked upregulation of inflammation‐associated regulatory networks (Figure S23, Supporting Information). Moreover, the percentage of M1 and M2 phenotype macrophages in each group was quantified by scRNA‐seq, which demonstrated a significant upregulation in the number of M1 macrophages within the TME following cmExo^aCD11b^ treatment (Figure 5k). Finally, to confirm the scRNA‐seq immunophenotype findings, we performed flow cytometry analysis on mouse pancreatic tumor samples. The results confirmed that cmExo^aCD11b^ treatment significantly enhanced the expression of CD86 protein and downregulated the level of CD206 protein, indicating effective reversal of the iTME (Figure 5l–n). Collectively, these results suggest that cmExo^aCD11b^ effectively reprograms the TME by inducing the polarization of M2 macrophages into M1 phenotype, thereby reversing its immunosuppressive state.

### 2.5 cmExo^aCD11b^ Promotes Cytotoxic Effects CD8^+^ T Cells

**Figure 6:**
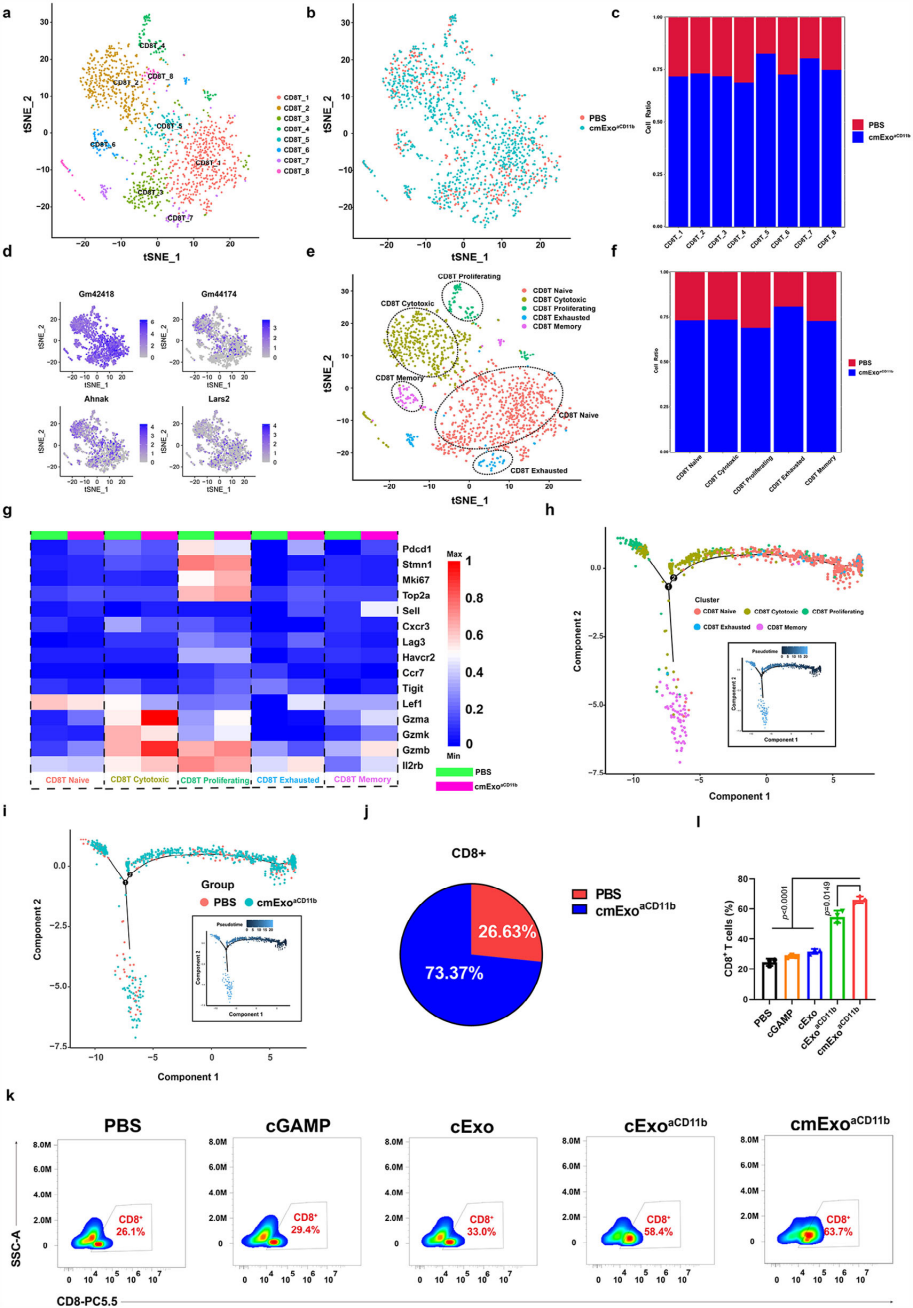
cmExo^aCD11b^ enhances CD8^+^ T cell functionality, promoting cytotoxic T lymphocyte (CTL) generation and immune memory formation in the TME. a) t‐SNE projection of CD8^+^ T cells, color‐coded by subpopulations. b) t‐SNE plot showing the separation of CD8T cell subpopulations between PBS and cmExo^aCD11b^ treatments. c) Proportional distribution of CD8T cell subpopulations (CD8T_1‐CD8T_8) following PBS or cmExo^aCD11b^ treatment. d) Gene expression patterns of Gm42418, Gm44174, Ahnak, Lars2 projected onto t‐SNE plots. e) t‐SNE plots showing CD8T cell subpopulations defined by distinct markers. f) Proportional distribution of CD8T cell subpopulation (CD8T Naïve, CD8T Cytotoxic, CD8T Exhausted, CD8T Memory, and CD8T Proliferating) under PBS or cmExo^aCD11b^ treatments. g) Heatmap of differentially expressed genes in CD8T cell subpopulations following PBS or cmExo^aCD11b^ treatment. h) Pseudotime trajectory mapping of CD8T subclusters (CD8T_1‐CD8T_8). i) Pseudotime trajectory mapping of CD8T subpopulations (PBS, cmExo^aCD11b^). j) Schematic representation of CD8^+^ T cell population changes under different treatment conditions, based on scRNA‐seq results. k) Representative flow cytometry results showing a significantly increase in CD8^+^ T cell infiltration in tumor issues after treating by cmExo^aCD11b^. l) Statistical analysis of the proportion of CD8^+^ T cells in mouse tumors. All data are presented as mean ± s.d. (*n* = 3); Statistical significance was calculated by one‐way ANOVA followed by Tukey’s multiple comparisons test.

In addition to its functions on macrophages, cmExo^aCD11b^ significantly expanded distinct subpopulations of CD8^+^ T cells infiltrating the TME, as revealed by scRNA‐seq analysis. CD8^+^ T cells were categorized into 8 distinct subcellular clusters (**Figure** **6**a). Compared to PBS group, all CD8^+^ T cell subpopulations were markedly increased following cmExo^aCD11b^ treatment (Figure 6b,c). Notably, CD8T_5 subcluster demonstrated the most significant activation upon cmExo^aCD11b^ induction. Within this subpopulation, genes such as Gm42418, Gm44174, Ahnak, and Lars2, which are associated with enhanced cell activation, migration, and effector functions, were highly expressed (Figure 6d). Moreover, based on the functional marker genes of CD8^+^ T cells, these cells were further categorized into five classes: CD8T Naïve (Ccr7, Lef1, Sell), CD8T Cytotoxic (Gzma, Gzmb, Gzmk), CD8T Exhausted (Lag3, Pdcd1, Havcr2, Tigit), CD8T Memory (Il2rb, Cxcr3, Gzma), and CD8T Proliferating (Mki67, Top2A, Stmn1) (Figure 6e). Similarly, in the reclassified subgroups, all subpopulation were also upregulated following cmExo^aCD11b^ induction (Figure 6f). Next, we generated a heatmap to visualize the highly expressed genes in the CD8T subpopulation between the PBS and cmExo^aCD11b^ groups (Figure 6g). Notably, Gzma, Gzmb, and Il2rb were highly expressed in the CD8T Cytotoxic subpopulation after cmExo^aCD11b^ induction. These genes are involved in cell killing and proliferative capacity, and their elevated expression reflects a strong activation and enhanced cytotoxicity of CD8T Cytotoxic cells. Similarly, Mki67, a gene associated with cell proliferation, was also significantly upregulated upon cmExo^aCD11b^ induction, suggesting higher levels of cell cycle‐related transcript expression. Furthermore, we applied Monocle2 to analyze CD8^+^ T cells, revealing a trident trajectory map (Figure 6h,i). Consistent with the t‐SNE subpopulation analysis, most CD8T Naïve cells were found to occupy the initial stage of the trajectory. As the trajectory progresses, CD8T cells gradually transition to the CD8T Cytotoxic phenotype, which is responsible for eliminating tumor cells. Ultimately, this population differentiates into two branches: CD8T Proliferating and CD8T Memory. From these findings, we infer that CD8^+^ T cells within the TME play a critical role in tumor eradication following their conversion into cytotoxic T lymphocytes. Subsequently, these cells differentiated into CD8T cell proliferating and CD8T cell memory subsets, which collectively facilitate the amplification and establishment of CD8^+^ T cell immune memory. Finally, to validate the immunophenotype identified by scRNA‐seq, we employed flow cytometry to detect the expression of CD8^+^ T cells in tumors and spleen. The results were consistent with those obtained from scRNA‐seq (Figure 6j–l; Figure S24, Supporting Information). Based on these findings, we can conclude that cmExo^aCD11b^ enhances the expression of CD8^+^ T cells as well as their killing ability, which in turn modulates the TME.

### 2.6 cmExo^aCD11b^ Regulates CD4^+^ T Cells and DCs to Enhance Immune Response

**Figure 7:**
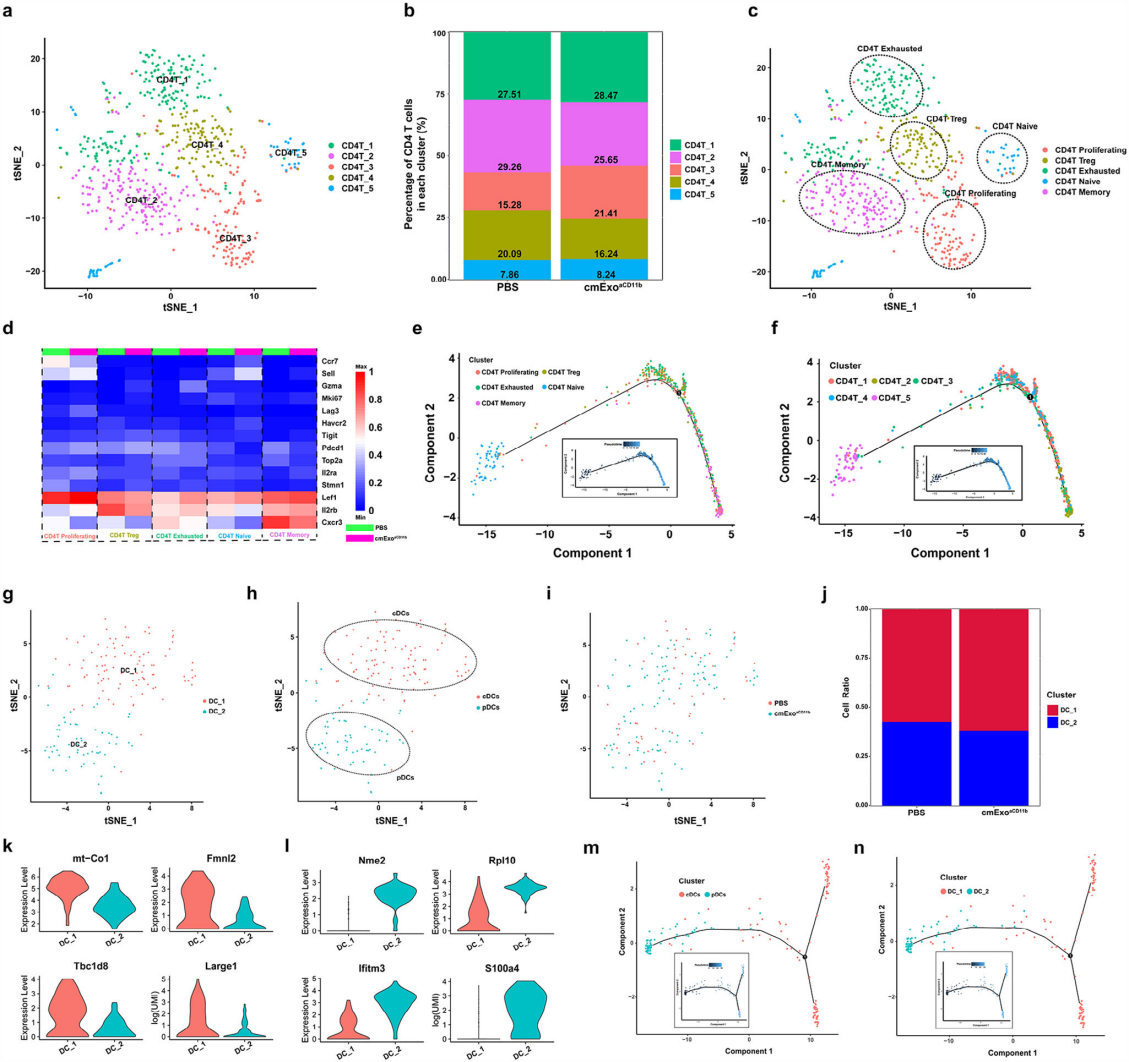
cmExo^aCD11b^ modulates functional CD4^+^ T cells and enhances the antigen‐presenting capability of DCs. a) t‐SNE projection map showing different color‐coded CD4^+^ T cell subpopulations. b) Percentage of CD4T_1 to CD4T_5 in PBS and cmExo^aCD11b^ treatment groups. c) t‐SNE plots showing CD4^+^ T cell subpopulations defined by distinct markers. d) Heatmap illustrating gene expression changes in different subpopulations following PBS and cmExo^aCD11b^ treatment. e. Pseudotime trajectory analysis of CD4T subpopulations (CD4T Exhausted, CD4T Memory, CD4T Treg, CD4T Naïve, CD4T Proliferating). f) Pseudotime trajectory analysis of CD4T subclusters (CD4T_1‐CD4T_5). g) t‐SNE projection map displaying different color‐coded DCs expressing H2‐Aa and Itgax. h) t‐SNE plots showing cell subpopulations defined by distinct markers. i) t‐SNE plot of all cells, color‐coded by treatment. j) The percentage of DC_1 to DC_2 in PBS and cmExo^aCD11b^ treatment groups. k,l) Violin diagram highlighting the top differentially expressed genes in DC_1 and DC_2 subpopulations. m) Pseudotime trajectory analysis of DCs subclusters (pDCs, cDCs). n) Pseudotime trajectory analysis of DCs subclusters (DC_1 to DC_2). All data are presented as mean ± s.d. (*n* = 3); Statistical significance was calculated by Student's t‐test or one‐way ANOVA followed by Tukey’s multiple comparisons test.

To investigate the role of CD4^+^ T cells within the TME, we also performed scRNA‐seq analysis of CD4^+^ T cells (Cd4). The scRNA‐seq results identified 5 distinct CD4^+^ T cell subpopulations (**Figure** **7**a). Notably, the CD4T_4 and CD4T_2 subpopulations were significantly downregulated, whereas the CD4T_3 subpopulations were upregulated. The remaining subpopulations were unaffected following cmExo^aCD11b^ induction (Figure 7b). These findings suggest that cmExo^aCD11b^ mainly modulates CD4^+^ T cells function through its effects on the CD4_2, CD4_3, and CD4_4 subpopulations. Next, CD4^+^ T cells were further classified into 5 subpopulations based on functional marker gene: CD4T Exhausted (Lag3, Pdcd1, Havcr2, Tigit), CD4T Memory (Il2rb, Cxcr3, Gzma), CD4T Treg (CTLA4, Foxp3, Il2ra), CD4T Naïve (Ccr7, Lef1, Sell), and CD4T Proliferating (Mki67, Top2A, Stmn1) (Figure 7c). By comparing Figure 7a,c, and analyzing the spatial location of subpopulations and their marker genes in the t‐SNE map, CD4T_4 was identified as a CD4T Treg subpopulation. This finding suggests that the cmExo^aCD11b^ treatment can reduce the abundance of CD4T Treg in the TME. Furthermore, CD4T Treg modulates Treg function by altering the expression of Il2rb and cxcr3 following cmExo^aCD11b^ induction (Figure 7d). The reduced expression of these genes inhibited the production of CD4T Treg, thereby mitigating Treg‐induced immunosuppression. Correspondingly, CD4T_3, was identified as CD4T Proliferating subpopulation. This subpopulation enhanced CD4^+^ T cell proliferation by upregulating the expression of Lag3, Il2ra, and Il2rb after cmExo^aCD11b^ induction (Figure 7d), thereby improving immune surveillance and facilitating a more effective attack on tumor cells. Similarly, CD4T_2 was identified as CD4T Memory subpopulation. These results suggest that cmExo^aCD11b^ treatment led to a reduction in CD4T Memory cell population, potentially due to the expansion of the immune response. Furthermore, we applied Monocle2 to analyze the temporal trajectory of CD4^+^ T cells. The analysis showed that most CD4T naïve cells reside at the initial stage of differentiation. As the trajectory progresses, these cells gradually differentiate into CD4T Tregs and CD4T exhausted cells, ultimately transforming into CD4T memory cells (Figure 7e,f). These results indicate that cmExo^aCD11b^ induction alleviates the depletion and functional inhibition of CD4^+^ T cells, facilitates the formation of CD4T memory cells, and mitigates the immunosuppressive effects of Tregs on anti‐tumor immunity. Additionally, scRNA‐seq results showed an upward trend in the number of CD4^+^ T cells following cmExo^aCD11b^ induction. Consistent with these results, flow cytometry analysis of CD4^+^ T cells in mouse tumors and spleens confirmed a similar increase, further validating the impact of cmExo^aCD11b^ on CD4^+^ T cell dynamics (Figure S25, Supporting Information).

To investigate the mechanism of action of DCs after cmExo^aCD11b^ induction, we also conducted scRNA‐seq analysis to investigate the phenotypic changes in DCs (H2‐Aa, Itgax). DCs were categorized into 2 subpopulations, DC_1 and DC_2, which were further defined as plasmacytoid DCs (pDCs: Siglech, Ccr9, Bst2) and conventional DCs (cDCs: Xcr1, Fscn1, ccl22) based on functional marker genes (Figure 7g,h). The scRNA‐seq analysis showed distinct clustering patterns between the PBS and cmExo^aCD11b^ treatment groups, highlighting the impact of cmExo^aCD11b^ on DC subpopulation dynamics (Figure 7i). By comparing Figure 7g,h, DC_1 was identified as cDCs, while DC_2 was identified as pDCs. Following cmExo^aCD11b^ induction, the populations of DC_1 showed an increasing trend, enhancing antigen presentation and T cell activation. This improvement highlights the crucial role of cDCs in initiating and regulating adaptive immune responses (Figure 7j). DC_1 enhances the antigen presentation ability of cDCs by modulating effector molecules such as mt‐Co1, Fmnl2, Tbc1d8, and Large1, thereby contributing to tumor cell elimination (Figure 7k). In contrast, DC_2 regulates immune responses by modulating effector molecules such as Nme2, Rpl10, Ifitm3, and S100a4, which activate pDCs and promote the production of IFN (Figure 7k,l). Further analysis of DCs using Monocle2 revealed a gradual transformation from pDCs to cDCs (Figure 7m,n). Based on these results, we infer that DCs rapidly produce substantial amounts of IFNs within the TME upon recognizing viral components. The production of IFN further facilitates the differentiation of pDCs into cDCs, which subsequently exhibit enhanced capabilities in antigen uptake, processing, and presentation. These activated cDCs effectively stimulate T cells, initiating specific immune responses and achieving the critical immunological functions of DCs.

### 2.7 The Therapeutic Efficacy of cmExo^aCD11b^ in PDX Mice

**Figure 8:**
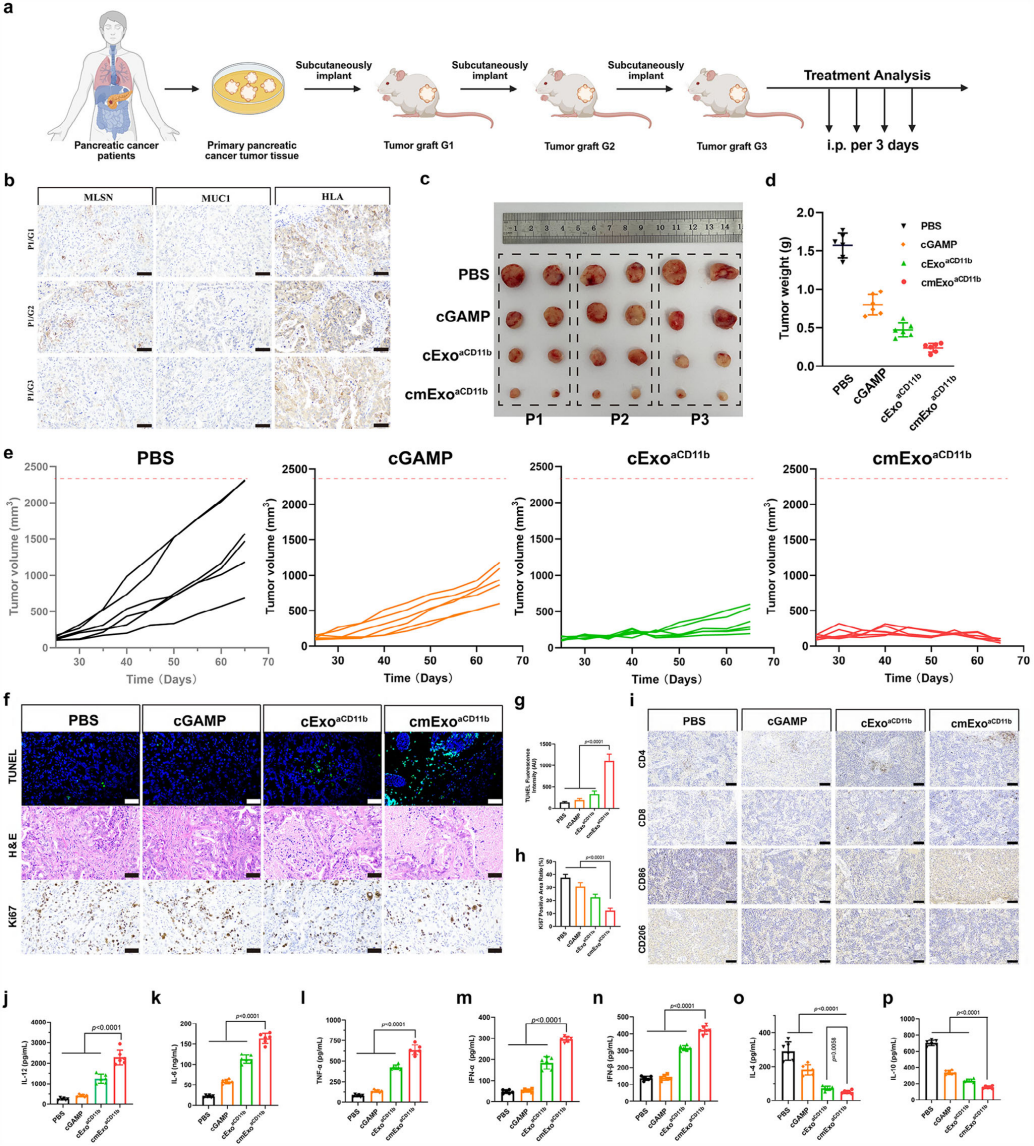
Therapeutic efficacy of cmExo^aCD11b^ in PDX pancreatic cancer models. a) Schematic representation of the PDX pancreatic cancer model construction. b) IHC staining of tumor tissues at different generations, demonstrating high expression of pancreatic cancer marker proteins MLSN and MUC1, as well as the humanized marker protein HLA. Scale bar: 100 µm. c) Tumor sizes in PDX mice across different treatment groups after 65 days of administration (*n* = 6, biologically independent samples). d,e) Tumor weight (d) and volume (e) of PDX mice after 65 days of treatment (*n* = 6, biologically independent samples). f) Therapeutic efficacy of cmExo^aCD11b^ in PDX pancreatic cancer mice, evaluated using TUNEL, H&E, and Ki67 staining (*n* = 3). Scale bar: 100 µm. g,h) Quantitative analysis of TUNEL fluorescence intensity and Ki67‐positive expression levels in tumor tissues across experimental groups. i) IHC analysis of CD4, CD8, CD86, and CD206 expression in PDX model spleen tissues. Scale bar: 100 µm. j–p) Cytokine levels of IL‐6, IL‐4, IL‐10, IL‐12, TNF‐α, IFN‐α, and IFN‐β in blood serum, detected by ELISA (*n* = 6). All data are presented as mean ± s.d.; statistical significance was calculated by one‐way ANOVA followed by Tukey’s multiple comparisons test (d, g, h, j‐p).

Based on the above results, cmExo^aCD11b^ showed an excellent therapeutic effect for pancreatic cancer therapy. To simulate clinical conditions, a human pancreatic cancer PDX tumor model was further constructed. The construction process of the PDX model and the dosing process are illustrated in **Figure** **8**a. During the establishment of PDX model, IHC staining of the tumor tissues at all stages was performed. The results showed that pancreatic cancer signature proteins, including MLSN and MUC1, were highly expressed in all three generation tumors (G1‐G3) from three patients (P1‐P3). In addition, all samples exhibited high expression of the humanized protein HLA, indicating the retention of human tissue characteristics and confirming the successful establishment of the PDX model (Figure 8b; Figure S26, Supporting Information). Once the tumor volume in the PDX mice reached ≈100 mm^3^, peripheral blood mononuclear cells (PBMCs) obtained from healthy human were injected into these mice to reconstruct the TME. Treatment was initiated when CD45^+^ cells accounted for ≈30% of the total PBMCs population, typically achieved within one week (Figure S27, Supporting Information). From this point, treatment was administered every 3 days thereafter. At 65 days post‐initial treatment, the cmExo^aCD11b^ group showed the most significant therapeutic efficacy compared to the other treatment groups (Figure 8c–e). Meanwhile, H&E staining of the major organs, including the heart, liver, spleen, lung and kidney revealed no tissue damage after treatment, indicating the high safety profile of cmExo^aCD11b^ (Figure S28, Supporting Information). Histopathological evaluations, including H&E staining and TUNEL assay of tumor sections, revealed extensive necrotic regions in the cmExo^aCD11b^ treatment group. Furthermore, cmExo^aCD11b^ significantly inhibited the expression of Ki67, confirming the anti‐tumor effect of cmExo^aCD11b^ (Figure 8f–h). In addition, IHC staining of spleen showed that the CD4^+^ and CD8^+^ T cells were elevated in the cmExo^aCD11b^ group (Figure 8i). M1‐phenotype macrophages are increased in the mouse spleen (Figure 8i). The levels of IL‐12, IL‐6, TNF‐α, IFN‐α, and IFN‐β were significantly elevated, while IL‐4 and IL‐10 levels were decreased in the peripheral blood of mice in the cmExo^aCD11b^ group, further confirming the activation of immune response by cmExo^aCD11b^ in vivo (Figure 8j–p). Together, all these results, in consistence with data from orthotopic pancreatic cancer mouse model, further confirmed the therapeutic efficacy of cmExo^aCD11b^ for pancreatic cancer immunotherapy.

## 3 Discussion

In this study, we developed a novel Exo‐based drug delivery platform, named cmExo^aCD11b^, which effectively encapsulates high copy numbers of IL‐12 mRNA and the STING agonist cGAMP. Surface modification with aCD11b enabled its preferential internalization by macrophages, leading to the activation of the STING signaling cascade, repolarization of macrophages, and remodeling of the TME. Collectively, these combined effects alleviated immunosuppression and inhibited tumor growth.

STING pathway activation has been reported to upregulate PD‐L1 expression on tumor cells, potentially diminishing therapeutic efficacy by enhancing immune checkpoint signaling.^[^ ^3^, ^23^, ^24^ ^]^ To counter this, PD‐L1 antibodies are commonly used to block immune checkpoints and prevent immune escape. For example, Gao et al. demonstrated that MnTe₂NSs combined with PD‐L1 antibodies effectively inhibited tumor growth.^[^ ^25^ ^]^ Similarly, Li et al. reported enhanced anti‐breast cancer efficacy when PD‐L1 blockade was paired with STING activation strategies.^[^ ^5^ ^]^ Despite these advances, PD‐L1 blockade is not universally effective across all cancer types. Clinically, some patients exhibit primary resistance, possibly due to a highly iTME or a low tumor mutational burden.^[^ ^26^, ^27^ ^]^ Moreover, even in initially responsive tumors, acquired resistance can develop via alternative immune escape pathways such as TIM‐3 and LAG‐3.^[^ ^28^, ^29^, ^30^, ^31^, ^32^ ^]^ Tumor heterogeneity further complicates treatment, as spatially variable PD‐L1 expression within tumors may impair therapeutic efficacy.^[^ ^33^, ^34^ ^]^ In addition, systemically administered PD‐L1 antibodies often suffer from poor targeting specificity, leading to rapid clearance by the RES. This not only increases therapeutic costs but also reduces bioavailability and limits therapeutic efficacy. Consequently, while combining PD‐L1 antibodies with STING agonists shows promise, it may not always represent the most optimal strategy or broadly applicable strategy for combating tumor progression. To address the challenges mentioned above, we employed the cmExo^aCD11b^ platform to reprogram the TME and inhibit tumor immunosuppressive state. Notably, we administered a relatively low dose of cGAMP (5 µg per mouse), which is substantially lower than doses typically used in previous studies (e.g., 100 µg per mouse).^[^ ^5^ ^]^ Furthermore, the introduction of IL‐12 mRNA enhanced the functional efficacy of the STING pathway. As expected, scRNA‐seq data revealed that cmExo^aCD11b^ effectively reprograms macrophages within the TME. Notably, while an increase in Mac_6 cells—a subset associated with M2‐like macrophages‐was observed, their relative proportion remained small and did not impede the overall increase in M1‐like macrophages. These results suggest that cmExo^aCD11b^ primarily facilitates the polarization of M2 macrophages to M1 macrophages by regulating the Mac_1 and Mac_4 clusters, thereby reversing the iTME and enhancing immune responses. To corroborate these findings, we investigated the phenotypic and gene expression changes in CD8^+^ T cells. Specifically, we focused on the expression of Gzma and Gzmb, as these molecules are key effector proteins in cytotoxic T lymphocytes.^[^ ^21^, ^35^ ^]^ Our result revealed a significant upregulation of Gzmb and Gzma within the CD8^+^ T Cytotoxic subpopulation, indicating that these cells are in an activated state. Interestingly, all five CD8^+^ T cell subpopulations classified based on their function were significantly increased following cmExo^aCD11b^ treatment. These results further suggest that cmExo^aCD11b^ treatment not only reprograms the macrophage population but also boosts the cytotoxic potential of CD8^+^ T cells by remodeling the TME. Finally, to evaluate the clinical applicability of the cmExo^aCD11b^ platform, we employed a PDX model of pancreatic cancer, which better recapitulates the complexity of human tumors. Treatment with cmExo^aCD11b^ significantly inhibited tumor growth in this model, further confirming its therapeutic potential. These results underscore the robust immunomodulatory and anti‐tumor capabilities of the cmExo^aCD11b^ platform in both murine and PDX pancreatic cancer model, providing valuable insights for the clinical exploration of STING pathway‐based immunotherapies.

Despite the promising therapeutic results demonstrated by the cmExo^aCD11b^ platform, several limitations must be addressed to facilitate its clinical translation. First, the drug‐loading capacity of cmExo^aCD11b^ must be improved, as the current drug loading efficiency of cGAMP into Exos is only 3.06%. This low efficiency requires a greater quantity of Exo and thereby prolongs the preparation time for the platform. Improving the encapsulation strategy will be crucial for enhancing scalability and efficiency. Furthermore, our drug delivery system utilizes CD11b antibody modification to enhance targeting of macrophages, with the primary goal of promoting uptake by M2 macrophages and inducing their repolarization into the tumor‐suppressive M1 phenotype. However, since CD11b is highly expressed on both M1 and M2 macrophages, cmExo^aCD11b^ is likely to be internalized by both subtypes. Indeed, previous studies have shown that CD11b‐based delivery system targets both M1 and M2 macrophage populations.^[^ ^36^ ^]^ Fortunately, our experimental data showed no treatment‐related adverse effects despite significant cmExo^aCD11b^ internalization by M1 macrophages. Nonetheless, we recognize the importance of further exploring the differential responses of M1 versus M2 macrophages to cmExo^aCD11b^, as such findings may unveil macrophage‐specific therapeutic strategies in cancer immunotherapy.

Second, our findings showed increased expression of PD‐1 on CD8⁺ T cells and PD‐L1 on tumor tissues following treatment with cmExo^aCD11b^, as assessed by immunofluorescence. Studies have shown that IL‐12 can indirectly regulate the PD‐1/PD‐L1 pathway via IFN‐γ, and the upregulation of this pathway promotes tumor immune escape.^[^ ^37^ ^]^ Despite the observed PD‐1 upregulation, the cmExo^aCD11b^ platform demonstrated strong immunostimulatory effects, which we attribute to its ability to reprogram TME. The cmExo^aCD11b^ platform delivers both cGAMP and IL‐12 mRNA, which together initiate a coordinated immune activation cascade: 1) M2‐to‐M1 repolarization: scRNA‐seq analysis revealed significant reprogramming of macrophage subpopulations, with increased representation of M1‐like Mac_2, Mac_3 and Mac_5 clusters, and decreased representation of M2‐like Mac_1 and Mac_4 clusters. 2) M1 macrophage activation: Upon activation, M1 macrophages robustly secrete pro‐inflammatory cytokines such as TNF‐α, IL‐12, and IL‐6, which promote Th1 cell differentiation and augment IFN‐γ production by CD8⁺ T cells, establishing a positive feedback loop of immune activation.^[^ ^38^ ^]^ 3) T cell recruitment and priming: M1 macrophages also secrete chemokines CXCL9 and CXCL10, which recruit CD8⁺ T cells into the TME. Meanwhile, ATP released by these macrophages activates the NLRP3 inflammasome, thereby driving DC maturation and further T cell priming. Collectively, these activities position M1 macrophages as a key orchestrator in dismantling immunosuppressive networks within the TME.^[^ ^39^ ^]^ Moreover, our scRNA‐seq results indicated a marked increase in CD8^+^ T cell infiltration and a concomitant reduction in Tregs within the CD4^+^ T cell compartment, reinforcing the anti‐tumor immune response. In light of these results, we conclude that while PD‐1 expression on CD8⁺ T cells was elevated—possibly as a feedback mechanism in response to increased immune activation—the overall TME was shifted toward a more immunostimulatory and antitumor state. Therefore, further exploring the immunotherapy mechanisms of cmExo^aCD11b^ for pancreatic cancer is significantly important.

Third, while our results provide compelling preclinical data, further investigations are essential to fully explore the clinical potential of the cmExo^aCD11b^ platform. In the PDX model, although cmExo^aCD11b^ has considerable therapeutic effect, we found that the expression of some immune cells, such as CD4^+^ T cells, CD8^+^ T cells, etc., was low in tumor tissues. To better understand the immune landscape in vivo in PDX model mice, we performed IHC on mouse spleens to analyze the changes in the expression of CD4, CD8, CD86, and CD206. The spleen samples showed increased infiltration of CD4^+^ and CD8^+^ T cells as well as M1 phenotype macrophages, suggesting a systemic immune activation despite the limited local infiltration observed within the tumor. Interestingly, preliminary studies showed that even with low intratumoral immune cells, tumor volume still decreased over time. To investigate the role of the TME in this tumor reduction, we propose applying scRNA‐seq to gain cell‐type‐resolved insights into TME remodeling in the PDX model. Meanwhile, while our current study focuses on pancreatic cancer, it is essential to evaluate the generalizability and therapeutic efficacy of the cmExo^aCD11b^ platform in other cancer models. Addressing its performance across diverse cancers will offer profound insights into the adaptability and broader applicability of this platform. Last but not least, translating cmExo^aCD11b^ into clinical application will require rigorous optimization of its pharmacokinetics, biosafety, and large‐scale production methodologies. Rigorous preclinical studies, including long‐term assessments of immune memory and tumor recurrence, will be essential to validate the durability and safety of the cmExo^aCD11b^ platform. In conclusion, the cmExo^aCD11b^ platform facilitates the polarization of M2 macrophages into M1 phenotype within the TME, resulting in the secretion of pro‐inflammatory cytokines. To the best of our knowledge, this research represents the first integration of nucleic acid‐based therapy with STING pathway activation to modulate the TME and suppress tumor progression. We believe the cmExo^aCD11b^ platform serves as a strong foundation for the development of next‐generation cancer immunotherapy.

## 4 Experimental Section

### Materials

Dulbecco's Modified Eagle Medium (DMEM), Roswell Park Memorial Institute 1640 Medium (RPMI‐1640), and penicillin/streptomycin‐glutamine (100 x) were obtained from Gibco (Waltham, MA, USA). Leibovitz's L‐15 Medium (L15) and fetal bovine serum (FBS) were acquired from Procell Life Science & Technology Co., Ltd (China). cGAMP was supplied by Shanghai Taojutsu Biotechnology Co., Ltd (China). IL‐12 mRNA plasmids (Human IL‐12 mRNA plasmid, gene ID: 3592; and Murine IL‐12 mRNA plasmid, gene ID: 16 159) were obtained from Yanming Biotechnology Co., Ltd (China). Cytochalasin D, nystatin, sucrose, phosphate‐buffered saline (PBS), PKH26, and PKH67 were obtained from Sigma‐Aldrich (St. Louis, MO, USA). The TransExoTM Serum/Plasma Exosome Total RNA Extraction Kit and PrimeScript RT Master Mix were obtained from TransGen Biotechnology Co., Ltd (China). The CD11b antibody (catalog number 101247) was purchased from Biolegend (USA). The BCA protein assay kit was sourced from Thermo Fisher Scientific (USA). All flow antibodies, including Fixable Viability Dye eFluor 780, anti‐CD45‐KO525, anti‐CD8‐eFluor 710, anti‐CD4‐APC, anti‐CD86‐PE, anti‐CD206‐APC, anti‐F4/80‐PacBlue and anti‐CD11b‐FITC were purchased from Thermo Fisher Scientific (USA). The anti‐TSG101, anti‐Alix, and anti‐CD9 antibodies were purchased from Abcam (USA). The anti‐β‐Actin, anti‐GAPDH, anti‐CD86, anti‐CD206, anti‐IRF3, anti‐IKKα, anti‐IKKβ, anti‐IκBα, and anti‐phospho‐IKKα/β (Ser176/180) antibodies were purchased from BOSTER (China). The anti‐phospho‐IκBα (Ser32), anti‐CD63 antibodies and anti‐phospho‐IRF3 (Ser396/Ser386) antibodies were purchased from Affinity Biosciences (USA). The anti‐STING antibody was purchased from ABclonal (USA). The anti‐phospho‐STING (Ser365/Ser366) and NF‐κB p65 antibody was purchased from Cell Signaling Technology (USA). Phospho‐NF‐κB p65(Ser536) was purchased from Zenbio (USA). ELISA kits, including IL‐4, IL‐6, IL‐10, IL‐12, TNF‐α, IFN‐α, and IFN‐β, were purchased from Elabscience (China). The TransScript All‐In‐One First‐Strand cDNA Synthesis SuperMix for qPCR Kit and the 2× PerfectStart Green qPCR SuperMix were purchased from TransGen Biotechnology Co., Ltd (China).

### Cell Culture

MEF and RAW264.7 cells were all grown in DMEM supplemented with 10% FBS and 1% penicillin‐streptomycin‐glutamine under standard culture conditions. THP‐1 monocytes and Panc02 cells were propagated in RPMI‐1640 containing 10% FBS, 1% penicillin‐streptomycin‐glutamine, and 0.1% 2‐mercaptoethanol. Macrophage differentiation of THP‐1 cells was induced by introducing Phorbol 12‐myristate 13‐acetate (PMA, 200 nm). BMDMs were isolated from the femurs of C57BL/6J mice. BMDMs were cultured and stimulated with M‐CSF (50 ng mL^−1^). K210 cells were isolated from the spontaneous pancreatic ductal adenocarcinoma of C57BL/6J LSL‐Kras(G12D); Trp53 fl/fl; Pdx1‐Cre Mice. K210 and Luc‐K210 cells were cultured in DEME medium supplemented with 10% FBS and 1% penicillin‐streptomycin‐glutamine.

### Preparation and Characterization of cmExo^aCD11b^

First, mExo was prepared using CNP technology as previously described.^[^ ^18^ ^]^ The IL‐12 mRNA plasmid was transiently transfected into MEF cells utilizing CNP technology. The optimal conditions for transfection were set at: 200 V voltage, 10 ms, 5 pulses. At 24 h post‐transfection, mExo was extracted from the culture supernatant and isolated via differential centrifugation.^[^ ^40^ ^]^ Briefly, the cell culture medium was centrifuged at 300 g for 10 min to eliminate cells. Subsequently, a centrifugation step at 2,000 g for 10 min was performed to eliminate cell debris and apoptotic bodies. The sample was then centrifuged at 10,000 g for 20 min to remove large microvesicles. The resulting supernatant was subsequently filtered via 0.22 µm filter. These steps were conducted to ensure the effective separation and removal of various cellular components. Finally, mExo was precipitated using ultracentrifugation (100,000 g, 70 min) with a Beckman Coulter Optima MAX‐XP ultracentrifuge (USA). After that, the resulting pellet was resuspended in PBS. The ultracentrifugation process was repeated twice to ensure purity. All steps were conducted at a constant temperature of 4 °C to preserve Exo integrity. To obtain mExo conjugated with aCD11b, DSPE‐PEG2000‐NHS, and aCD11b (6:1, m:m) were initially reacted for 24 h at 4 °C to synthesize DSPE‐PEG2000‐aCD11b. To determine the optimal linkage ratio of mExo to DSPE‐PEG2000‐aCD11b, aCD11b‐PE (BioLegend) was coupled to DSPE‐PEG2000‐NHS and then determined the optimal linkage ratio of mExo to DSPE‐PEG2000‐aCD11b (0:1, 1:1, 1:2, 1:4, 1:6, 1:8, m:m) based on flow cytometry. mExo (concentration: 20 mg/mL) was coupled with DSPE‐PEG2000‐aCD11b at 37 °C for 1 h to yield mExo conjugated with aCD11b. Subsequently, the mixture was filtered through a 0.22 µm membrane and subjected to ultracentrifugation at 100,000 g for 2 h to remove unbound antibodies, yielding purified mExo^aCD11b^. Lastly, cGAMP was loaded into the mExo^aCD11b^ at a mass ratio (1:20, 1:10, 1:5, 2:5, 3:5, m:m) using sonication (sonication condition: 10 s, membrane repair 50 s, 10 cycles) to produce the final product (cmExo^aCD11b^). In addition, cExo^aCD11b^ was obtained by the above method, by combining an empty Exo‐modified CD11b antibody and loading cGAMP. Next, the structure and characterization of cmExo^aCD11b^ were evaluated. First, the protein concentration of cmExo^aCD11b^ was determined using the BCA assay method. The morphology of cmExo^aCD11b^ was visualized via Cryo‐TEM with a FEI Talos L120C microscope. The concentration of cmExo^aCD11b^ was measured using a NTA (Izon, New Zealand). The particle size and ζ‐potential of cmExo^aCD11b^ were analyzed using Dynamic Light Scattering instrument (DLS, Malvern ZS90, Malvern Panalytical Ltd, UK). Marker proteins of cmExo^aCD11b^, including CD9, CD63, Alix, and TSG101, were detected by Western blotting and nanoflow cytometry (NanoFCM, Xiamen, China). To evaluate the loading efficiency of cGAMP, high‐performance liquid chromatography (HPLC) analysis was conducted using a system from Waters Corporation system (Milford, MA, USA). The system was equipped with a C18 column (InertSustain AQ‐C18). The mobile phase consisted of methanol/water (10:90, v:v), the flow rate was 1.0 mL min^−1^ and the temperature was set at 25 °C. Samples were analyzed at 257 nm. cmExo^aCD11b^ was also obtained. In order to measure the load of cGAMP within Exo, cmExo^aCD11b^ was lysed using 10% Triton X100. Subsequently, the sample was centrifuged at 10,000 g for 10 min, and the concentration of cGAMP was measured in the supernatant using HPLC. The mass of cGAMP contained in cmExo^aCD11b^ was further calculated from the standard curve of cGAMP obtained from HPLC measurements, and finally the drug loading capacity (DL) of cmExo^aCD11b^ was calculated. Similarly, the cGAMP encapsulation efficiency (EE) was calculated according to Equations (**1**) and (2).

(1) $$\mathrm{DL}\%=\frac{m\left(cGAMP\right)}{m\left(cmExo^{aCD11b}\right)}$$

*m(cGAMP)* represents the quality of cGAMP loaded into mExo^aCD11b^.

*m(cmExo^aCD11b^)* represents the quality of cmExo^aCD11b^

(2) $$\mathrm{EE}\%=\frac{m\left(cGAMP\right)}{m\left(total\;cGAMP\right)}$$

*m(cGAMP)* represents the quality of cGAMP loaded into mExo^aCD11b^.

*m(total cGAMP)* represents the total input cGAMP quality.

Additionally, to analyze the loading efficiency of IL‐12 mRNA, total RNA was isolated from cmExo^aCD11b^ using the TransExoTM Serum/Plasma Exosomal Total RNA Extraction Kit. The extracted RNA was reverse transcribed using the First‐Strand cDNA Synthesis Kit. Expression levels of IL‐12 mRNA in cmExo^aCD11b^ and the U6 gene were evaluated by RT‐qPCR using SYBR Green PCR Master Mix. The human IL‐12 mRNA primers were as follows: forward, TGAGAGTTGCCTAAATTCCAGAGAG; reverse, CCACCTGGTACATCTTCAAGTCTTC. The mouse IL‐12 mRNA primers were as follows: forward, AAACCACCTCAGTTTGGCCA; reverse, TCTTCAGCAGGTTTCGGGA. The mouse U6 primers were as follows: forward, CTCGCTTCGGCAGCACA, reverse, AACGCTTCACGAATTTGCGT.

### The Cellular Uptake of cmExo^aCD11b^

To study the cellular uptake of cmExo^aCD11b^ by target cells, PKH26/67‐labeled cmExo^aCD11b^ was first constructed. Briefly, cmExo^aCD11b^ was mixed with PKH26/67 dye and dispersed in Diluent C for 5 min, followed by incubation at room temperature for 20 min. The mixture was centrifuged (100,000 g, 70 min) to remove the excess dye, and the final sample was stored at 4 °C. PKH26/67‐labeled cmExo^aCD11b^ (10 µg) was incubated with RAW264.7 cells or THP‐1 cells (1 × 10^4^ cells per well) for 4 h. After that, cells were washed three times with cold PBS and then fixed with 4% paraformaldehyde. The fluorescence intensity of the cells was analyzed utilizing flow cytometry (Beckman, USA). Each experimental group was repeated three times, and average fluorescence intensity was calculated for statistical analysis. Meanwhile, the cellular uptake of PKH67‐labeled cmExo^aCD11b^ was also recorded via a confocal laser scanning microscopy (FV‐3000, Olympus, Japan). To investigate the cellular uptake pathway of cmExo^aCD11b^ in RAW264.7 and THP‐1 cells, the following experimental procedure was performed: 1) cells were first incubated with Transferrin (0.1 mg mL^−1^), CT‐B (0.005 mg mL^−1^), and dextran (1 mg mL^−1^) (Invitrogen) for 1 h; 2) washed to remove unbound compound; 3) subsequently incubated with PKH67‐labeled cmExo^aCD11b^ for 4 h; and 4) finally visualized using confocal laser scanning microscopy.^[^ ^41^ ^]^ To investigate the endocytic mechanisms underlying cmExo^aCD11b^ internalization, pharmacological inhibitors targeting distinct uptake pathways—including nystatin (caveolae‐mediated), cytochalasin D (macropinocytosis), and sucrose (clathrin‐dependent)—were administered to recipient cells prior to treatment with cmExo^aCD11b^. Cellular internalization efficiency was subsequently quantified using flow cytometry, enabling assessment of the relative contribution of each pathway to Exo uptake.

### The Activation of cGAS‐STING Pathway

The activation of cGAS‐STING signaling pathway was evaluated by Western blotting. The expression of STING pathway‐related proteins, such as phospho‐STING (Ser365), phospho‐IRF‐3 (Ser396), phospho‐IKKα/β (Ser176/180), phospho‐IκBα (Ser32), and phospho‐NF‐κB p65 (Ser536), in RAW264.7/THP‐1 cells treated with cmExo^aCD11b^ for 6 h, was detected. Upon activation of the STING pathway, phospho‐IRF‐3 and phospho‐NF‐κB p65 undergo nuclear translocation, as observed by confocal laser scanning microscopy. Specifically, RAW264.7/THP‐1 cells at a concentration of 1 × 10^4^ cells were co‐cultured with cmExo^aCD11b^ for 6 h. Subsequently, their cell culture supernatants were harvested, and changes in IFN‐α and IFN‐γ levels were detected using the ELISA kit after different groups of administered treatments. For immunofluorescence staining, cells were cultured overnight at 4 °C with the primary anti‐phospho‐IRF‐3 antibody. After incubation, the cells were washed three times with cold PBS for 5 min. Cells were further incubated with FITC‐conjugated secondary antibody (green) for 1 h, followed by three more washes with PBS, each for 5 min. Finally, the nucleus was stained with Hoechst 33342 nuclear dye. Similarly, after overnight incubation with a primary anti‐phospho‐NF‐κB p65 antibody at 4 °C. The nuclei were incubated with Alexa Fluor 555‐conjugated secondary antibody (red) for 1 h, and the nucleus was stained with Hoechst 33342 nuclear dye in the same manner. The nuclear translocations of phospho‐IRF‐3 and phospho‐NF‐κB p65 was visualized through confocal laser scanning microscopy.

### The Activation of T Cells

To obtain T cells, spleens were carefully collected from different mouse models (CD4^+^ T cells from OT‐II mice, CD8^+^ T cells from OT‐I mice) and washed three times with sterile PBS. The spleens were ground and filtered using a 70 µm nylon cell strainer. Then, the suspension was centrifuged at 300 g for 5 min to obtain a single cell suspension. Sterile erythrocyte lysing buffer was mixed with the cell suspension and incubated at 25 °C for 10 min. The cell concentration was then adjusted to 2 × 10^8^ cells mL^−1^. Naïve CD8^+^ T cells, and naïve CD4^+^ T cells were collected according to the manufacturer's instructions (Elabscience, Product Number, MIM003N, MIM002N). BMDMs were incubated with PBS, cGAMP, cExo, cExo^aCD11b^, or cmExo^aCD11b^, respectively, followed by treatment with OVA peptide, OVA peptide‐conjugated MHC‐I (OVA 257–264) or MHC‐II (OVA 323–339) for 6 h to allow for antigen reconstitution and presentation. The cells were then incubated with OT‐I cells or OT‐II cells for 72 h. The OT cells were subsequently analyzed by flow cytometry to evaluate the proliferation rate of T cells.

### The Polarization of M2 Phenotype Macrophages

RAW264.7 cells or THP‐1 cells were seeded into 12‐well plates at 1 × 10^4^ cells per well and maintained at 37 °C for 24 h. Following treatment with cytokines (IL‐4 and IL‐10, each at 100 ng mL^−1^) for 24 h to induce M2 polarization, the culture medium was refreshed with complete DMEM medium. Subsequently, cmExo^aCD11b^ was introduced to the samples, and co‐incubated at 37 °C for 48 h. To assess whether M2 macrophages could be repolarized to an M1 phenotype, their morphology was first observed using confocal laser scanning microscopy. Subsequently, the secretion amounts of cytokines, including IL‐4, IL‐6, IL‐12, IL‐10, and TNF‐α, were detected in the cell supernatants using ELISA kits. Moreover, the level of CD86, CD206, and Arg‐1 proteins in the macrophages was detected by flow cytometry and Western blotting assay. For immunofluorescence analysis, RAW264.7 and THP‐1 cells were cultured and subsequently incubated overnight at 4 °C with a primary anti‐CD86 antibody. Following primary antibody incubation, cells were washed three times with cold PBS for 5 min each. Cells were then incubated with an Alexa Fluor 555‐conjugated secondary antibody (red) for 1 h, followed by three additional PBS washes (5 min each). Nuclei were counterstained with Hoechst 33342 nuclear dye. Similarly, for CD206 protein detection, cells were incubated overnight at 4 °C with a primary anti‐CD206 antibody, followed by incubation with a FITC‐conjugated secondary antibody (green) for 1 h. Nuclei were again counterstained with Hoechst 33342. The nuclear translocations of CD86 and CD206 were visualized using confocal laser scanning microscopy.

### The Therapeutic Effect for Pancreatic Cancer In Vivo

Male C57BL/6J mice (5–6 weeks old) were sourced from Beijing Huafukang Biological Technology Co., Ltd. (Beijing, China) and maintained for 7 days in a pathogen‐free facility with free access to water and food. Experimental protocols involving animals were conducted in compliance with Laboratory Animal Ethics Committee of the Cancer Hospital, Chinese Academy of Medical Sciences (approval No. NCC2023A090). First, an orthotopic pancreatic cancer mouse model was constructed. In detail, a small incision was created in the right upper abdomen of mouse to visualize healthy pancreatic tissue. Then, K210 cells (5 × 10^5^ cells) (tumor cells originating from the LSL‐Kras (G12D); Trp53fl/fl; Pdx1‐Cre mouse model) were inoculated into the healthy pancreas of the mice, and the wound was subsequently sutured. The orthotopic pancreatic cancer mice were then further fed. After 7 days, the mice were randomly divided into 5 experimental groups, each containing 6 mice (PBS, cGAMP, cExo, cExo^aCD11b^, and cmExo^aCD11b^). The drug dosage was standardized based on the cGAMP content, with each mouse receiving administration of 5 µg of cGAMP. In the meantime, drugs were administered every 3 days via intraperitoneal injection, and the tumor volume was monitored using an IVIS imaging system (PerkinElmer). After 45 days, mice were euthanized, and organs (heart, liver, spleen, lungs, and kidneys) along with tumors were harvested and paraffin‐embedded. Tissue sections underwent deparaffinized in xylene for 10 min, repeated three times, followed by rehydration in an ethanol gradient. Antigen retrieval and immunofluorescence staining or IHC staining were performed. For immunofluorescence staining: Green: Target protein labeled with primary antibody staining followed by FITC‐conjugated secondary antibody. Red: parallel labeling using primary antibody staining and Alexa Fluor 555‐tagged secondary antibody. Blue: Nuclei stained with 4′,6‐diamidino‐2‐phenylindole (DAPI). During the treatment period (24 h after drug administration), 100 µL of blood was collected from the orbital socket of each mouse, and serum was obtained by centrifugation (3,000 g, 10 min). The cytokine levels (IL‐4, IL‐6, IL‐10, IL‐12, TNF‐α, IFN‐α, and IFN‐β) in serum were analyzed by ELISA. Tumor or spleen specimens were mechanically disaggregated and enzymatically digested with collagenase IV (0.5 mg mL^−1^, 37 °C, 30 min). The digested homogenate was filtered through a 70 µm nylon mesh, followed by erythrocyte lysis for 10 min to generate single‐cell suspensions. Cell density was adjusted to 1 × 10^6^ ‐1 × 10^8^ cells mL^−1^ and used for following evaluation.

Cells were fixed and permeabilized to allow fluorescent probe entry for flow cytometry. Briefly, cells were first incubated with anti‐CD16/CD32 for 15 min to block nonspecific binding to Fc receptors. Cells were then stained with different antibodies, including Fixable Viability Dye eFluor 780, anti‐CD45‐KO525, anti‐CD8‐eFluor 710, anti‐CD4‐APC, anti‐CD86‐PE, anti‐CD206‐APC, anti‐CD11b‐FITC, or anti‐F4/80‐PacBlue. The corresponding experimental steps were performed according to the instructions.

### The Biosafety Assessment of cmExo^aCD11b^

In this work, the safety of cmExo^aCD11b^ was investigated through hemolysis experiments in vitro. Whole blood was obtained from murine retro‐orbital sinuses into sodium heparin‐coated anticoagulant tubes. Erythrocytes were obtained by centrifugation (3,000 rpm, 5 min) and subsequently diluted into a 2% erythrocyte suspension. Equal volumes of cmExo^aCD11b^ and the erythrocyte suspension were co‐incubated at 37 °C for 120 min. After centrifugation (3,000 rpm, 5 min), supernatants were transferred to a 96‐well plate, and absorbance at 545 nm was measured to calculate the hemolysis rates. Peripheral blood was collected from the mice to determine the variations in the levels of AST, ALT, BUN, and creatinine. In addition, H&E staining was used to examine the heart, liver, spleen, and lung in five groups, further assessing the safety of cmExo^aCD11b^.

### Single‐Cell RNA Sequencing Analysis

Raw sequencing data were processed using the 10x Genomics Cell Ranger pipeline (v.3.0.2) for de‐multiplexing, sequence alignment, quality control, and universal molecular identifiers (UMIs) quantification. Integrated datasets from distinct cellular subpopulation were generated to enable comparative transcriptomic profiling. Dimensionality reduction and visualization were performed via principal component analysis (PCA) and t‐SNE algorithms implemented in the Seurat package. For t‐SNE projections and clustering identification, the initial 18 principal components were obtained. Clusters containing cells with minimal or no expression of Gapdh and Eno1 (indicative of non‐cells) or those expressing markers exclusive to multiple cell types (indicative of doublets) were removed from further analysis. Distinct immune subsets were classified based on canonical marker expression: CD4^+^ T cells (Cd3e and Cd4), CD8^+^ T cells (Cd3e and Cd8a), DCs (H2‐Aa and Itgax), B cells (Cd79d and Ly6d), NK cells (Klrb1c), and macrophages (Adgre1 and Itgam). Cell cluster was confirmed by cross‐referencing with the ImmGen datasets. Cell proportions were normalized to library size to account for sequencing depth variability. Variations in gene expression among subclusters of macrophages, CD4^+^ T cells, CD8^+^ T cells, and DCs were examined using Loupe Browser v.5.0.0 and represented by their log2 fold change values (*p* < 0.05) for each treatment condition. The raw scRNA‐seq data had been deposited in the GEO database under the accession number GSE294507.

### The Therapeutic Effect for Pancreatic Cancer in PDX Models

Male NYG mice (2‐4 weeks old) were purchased from Liaoning Changsheng Biotechnology Co., Ltd. All experimental procedures were in accordance with ethical guidelines approved by Laboratory Animal Welfare Ethics Committee of Jilin University (approval No. SY202309047) as well as the Ethics Committee of the First Hospital of Jilin University (approval No. 23K241‐001). Written informed consent was acquired from all participating individuals prior to the study. Pancreatic cancer tissues from three patients were initially preserved in L15 medium and cut into pieces measuring 10–30 mm^3^ within 24 h. Subsequently, these tumor specimens were implanted subcutaneously into the right dorsal side of the NYG mice. Once the xenograft tumor volume reached 500–800 mm^3^, the tumor tissue was excised and immediately transplanted into the next generation of mice. Finally, when the xenograft tumors of the third‐generation mice reached ≈100 mm^3^, PBMCs isolated from healthy human were injected into these mice to reconstruct the TME. Treatment was initiated when CD45^+^ cells accounted for ≈30% of the total PBMCs population, typically achieved within one week. The mice were subjected to drug administration. They were randomly divided into four groups, each containing 6 mice (PBS, cGAMP, cExo^aCD11b^, and cmExo^aCD11b^). During the treatment period, drugs were administered every 3 days via intraperitoneal injection, and the administered dose was determined based on the amount of cGAMP (5 µg per mouse). The tumor volume was measured using dial calipers. Furthermore, the mice's vital signs and body weight were monitored and documented at intervals of 3 days. Same as above, during the treatment period (24 h after drug administration), 100 µL blood sample was drawn from the orbital sinus of each mouse, and the serum was isolated through centrifugation (3,000 g, 10 min). Changes in cytokines (IL‐12, IL‐4, IL‐10, IL‐6, TNF‐α, IFN‐α, and IFN‐β) in serum were analyzed by ELISA. Upon completion of the treatment phase, all mice were humanely sacrificed, and major organs (heart, liver, spleen, lung, and kidney), along with tumor tissues were excised and paraffin‐embedded for histological evaluation. The tissues were then subjected to H&E staining, IHC and immunofluorescence analysis.

### Statistical Analysis

The experimental data were obtained from a minimum of three independent replicates and were expressed as mean ± standard deviation (mean ± s.d.). Statistical significance was calculated by Student's t‐test or one‐way ANOVA followed by Tukey’s multiple comparisons test.

## Conflict of Interest

The authors declare no conflict of interest.

## Author Contributions

M.S., H.Z., and Y.M. contributed equally to this work. In this work, all authors contributed to the preparation of the manuscript and figures. Each author reviewed figures and approved the final version of the manuscript.

## Supporting information



Supporting Information

## Data Availability

The data that support the findings of this study are available from the corresponding author upon reasonable request.
