# Supplementary material for: In Situ Programming of the Tumor Microenvironment to Alleviate Immunosuppression for Pancreatic Cancer Immunotherapy
Source: Adv Sci (Weinh). 2025 Jun 23;12(34):e04008. doi: 10.1002/advs.202504008 (PMC12442614; doi:10.1002/advs.202504008)
Supplement: Supplementary file 1 — Supporting Information [file ADVS-12-e04008-s001.pdf]

ADVANCED SCIENCE
Open Access

Supporting Information

for *Adv. Sci.*, DOI 10.1002/advs.202504008

In Situ Programming of the Tumor Microenvironment to Alleviate Immunosuppression for Pancreatic Cancer Immunotherapy

Man Sun, Huan Zhang, Yarui Ma, Simiao Wang, Jiayi Chen, Yaxin Cui, Yun Zhang, Siyuan Hu, Dan Zhou, Pengchen Zhang, Yahui Liu, Betty Y.S. Kim, Wen Jiang, Xiaobing Wang and Zhaogang Yang**

Supporting Information

In situ programming of the tumor microenvironment to alleviate immunosuppression for pancreatic cancer immunotherapy

Man Sun[#], Huan Zhang[#], Yarui Ma[#], Simiao Wang, Jiayi Chen, Yaxin Cui, Yun Zhang, Siyuan Hu, Dan Zhou, Pengchen Zhang, Yahui Liu, Betty Y.S. Kim, Wen Jiang, Xiaobing Wang[*] and Zhaogang Yang[*]

M. Sun, H. Zhang, S. Wang, J. Chen, Y. Cui, Y. Zhang, S. Hu, P. Zhang, Z. Yang

School of Life Sciences, Jilin University, Changchun, 130012, China

E-mail: zhaogangyang@jlu.edu.cn

Y. Ma, X. Wang

State Key Laboratory of Molecular Oncology, National Cancer Center/National Clinical Research Center for Cancer/Cancer Hospital, Chinese Academy of Medical Sciences and Peking Union Medical College, Beijing, 100021, China

E-mail: wangxb@cicams.ac.cn

D. Zhou, Y. Liu

Department of Hepatobiliary and Pancreatic Surgery, The First Hospital of Jilin University, Changchun, 130021, China

B. Kim

Department of Neurosurgery, The University of Texas MD Anderson Cancer Center, Houston, TX, 77030, USA

W. Jiang

Department of Radiation Oncology, The University of Texas MD Anderson Cancer Center, Houston, TX, 77030, USA

~~E-mail: wjiang4@mdanderson.org~~

[#]These authors contributed equally

Keywords: Cancer immunotherapy, Pancreatic cancer, Exosomes, Drug delivery, cGAS-STING

Table S1. Drug loading capacity (DL) and encapsulation efficiency (EE) of cGAMP within cmExo^{aCD11b}, as determined by HPLC (n = 3).

**mExo^{aCD11b}:cGAMP(m:m)**	**20:1**	**10:1**	**5:1**	**5:2**	**5:3**
**DL (%)**	0.84 ± 0.04	1.98 ± 0.06	3.06 ± 0.04	2.98 ± 0.07	3.02 ± 0.06
**EE (%)**	15.86 ± 0.01	16.88 ± 0.02	17.30 ± 0.05	17.26 ± 0.02	17.32 ± 0.07

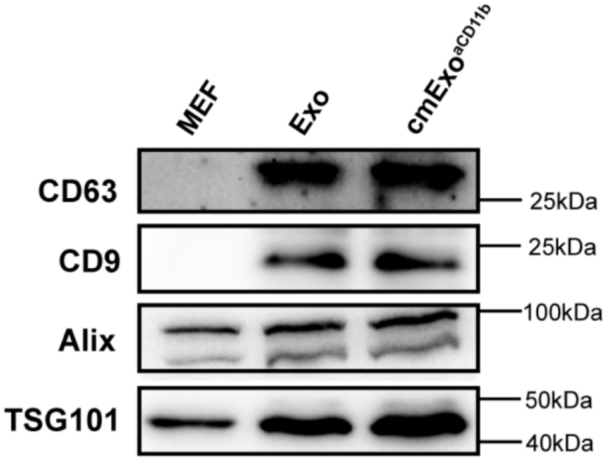

Figure S1. Signature proteins expressed on cmExo^{aCD11b}. Expression of signature proteins on Exo and cmExo^{aCD11b}, including CD9, CD63, TSG101, and Alix, as detected by Western blotting.

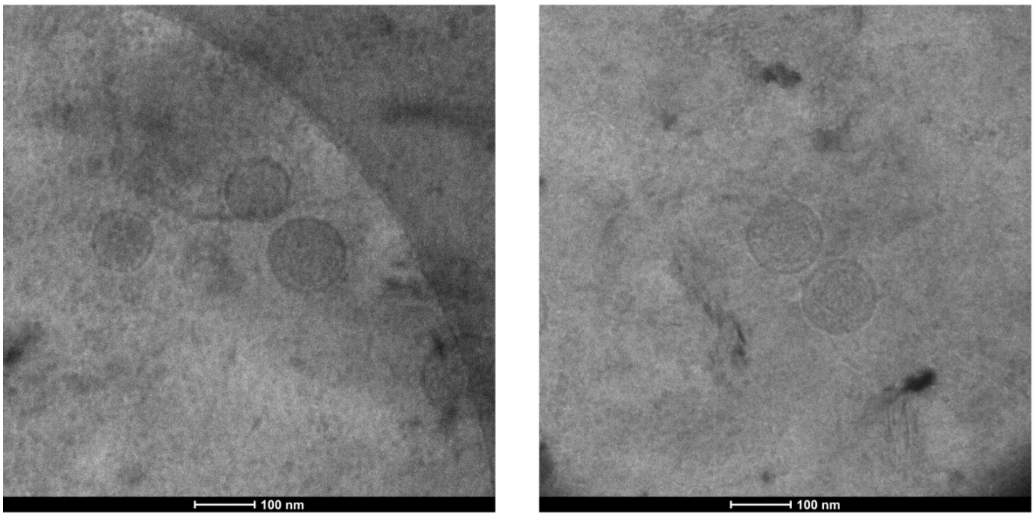

Exo **cmExo^{aCD11b}**

Figure S2. Morphology and distribution of Exo and cmExo^{aCD11b} observed by cryo-TEM. Cryo-TEM images showing excellent morphology and uniform size distribution of both Exo and cmExo^{aCD11b}. Scale bar: 100 nm.

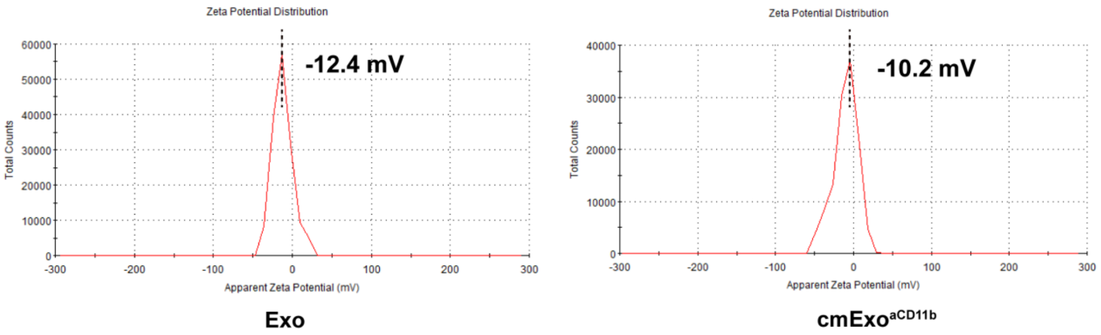

Figure S3. ζ-potential of Exo and cmExo^{aCD11b}, as detected by DLS (n = 3).

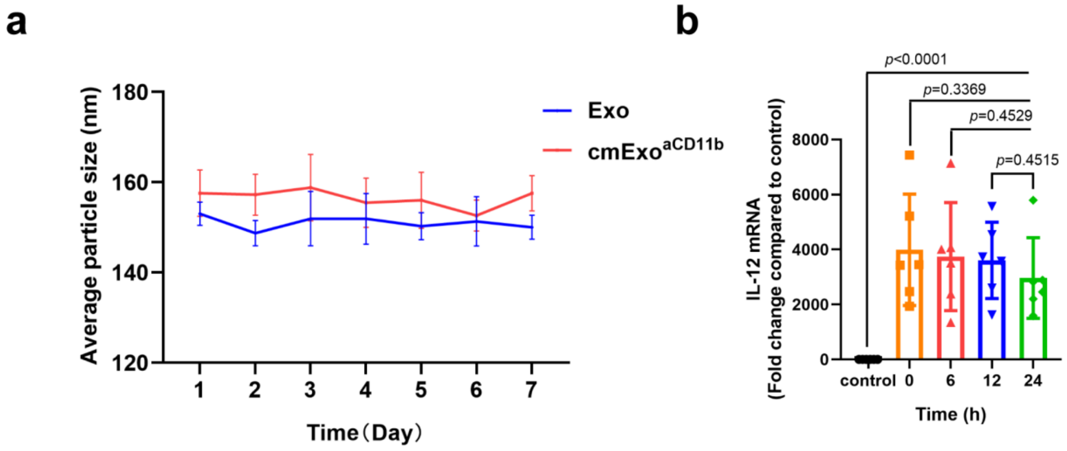

Figure S4. Stability of cmExo^{aCD11b}. a. Dynamic Light Scattering (DLS) analysis showing the size stability of Exo and cmExo^{aCD11b} after storage, confirming minimal changes in hydrodynamic diameter over time (n = 3). b. RT-qPCR analysis of IL-12 mRNA integrity in cmExo^{aCD11b} following 24 h incubation in whole blood. Results indicate that IL-12 mRNA remains stable and protected within the cmExo^{aCD11b} during systemic circulation (n = 6). Data are presented as mean ± s.d.; two-sided unpaired Student's t-tests were applied for comparisons.

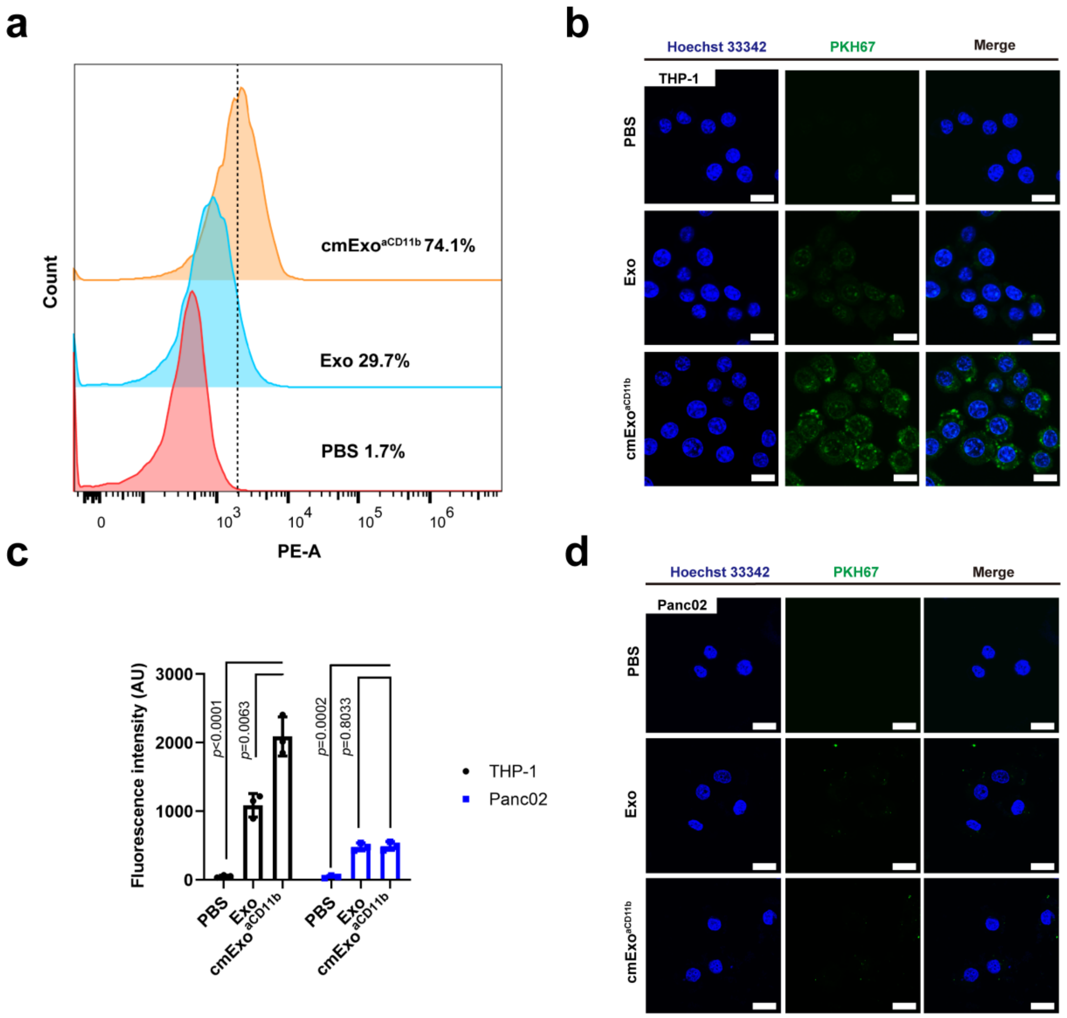

Figure S5. Cellular uptake of cmExo^{aCD11b} by different cell lines. a. Cellular uptake of cmExo^{aCD11b} by THP-1 cells, detected by flow cytometry. b. Cellular uptake of cmExo^{aCD11b} by THP-1 cells. Scale bar: 20 μm. c. Average fluorescence intensity of cmExo^{aCD11b} after uptake by different cells. d. Cellular uptake of cmExo^{aCD11b} by Panc02 cells. Scale bar: 20 μm. Data are presented as mean ± s.d.; two-sided unpaired Student's t-tests were applied for comparisons.

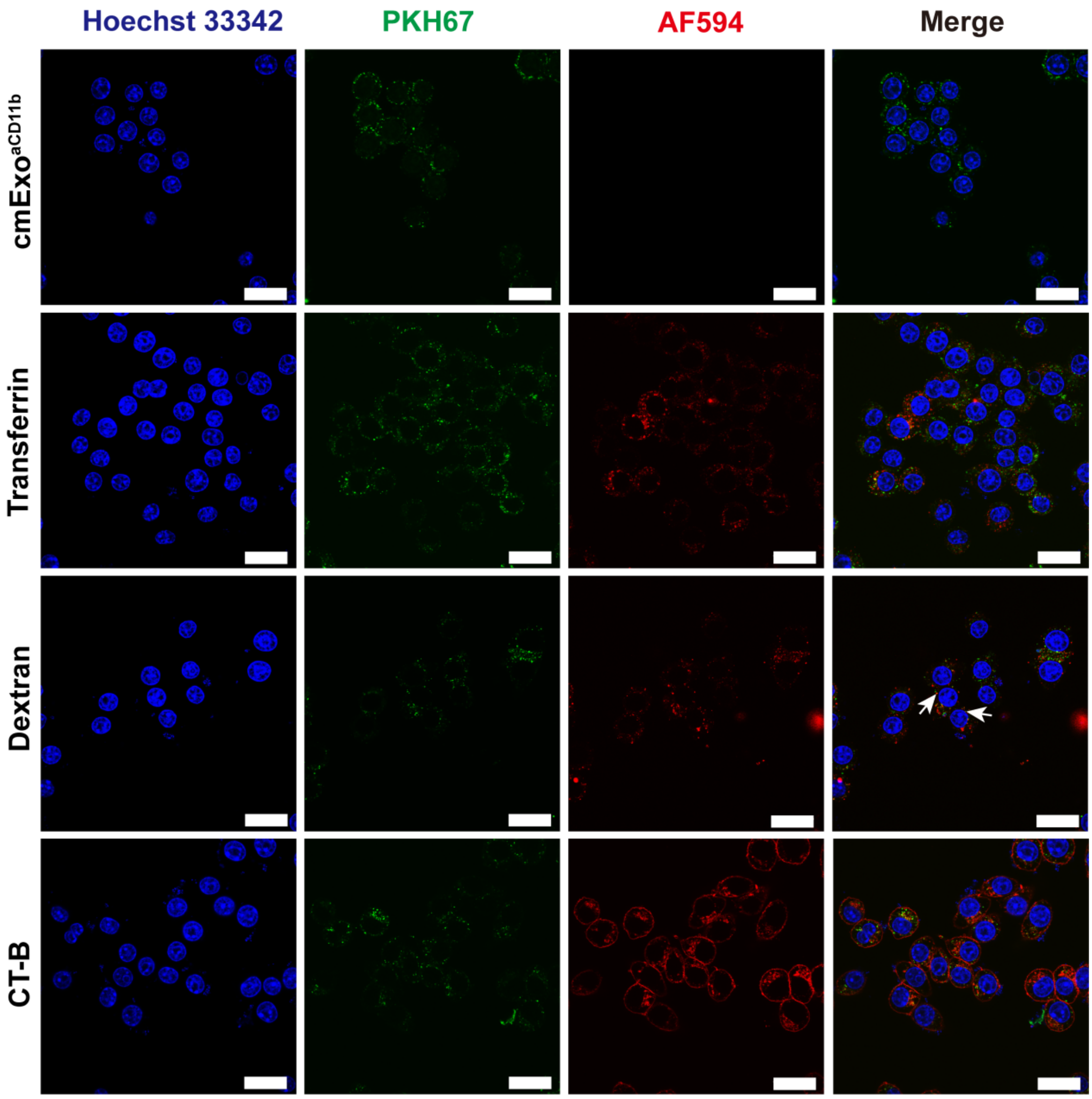

Figure S6. Cellular uptake mechanisms of cmExo^{aCD11b} by THP-1 cells. Arrow: cmExo^{aCD11b} co-localized with Dextran. Scale bar: 20 μm.

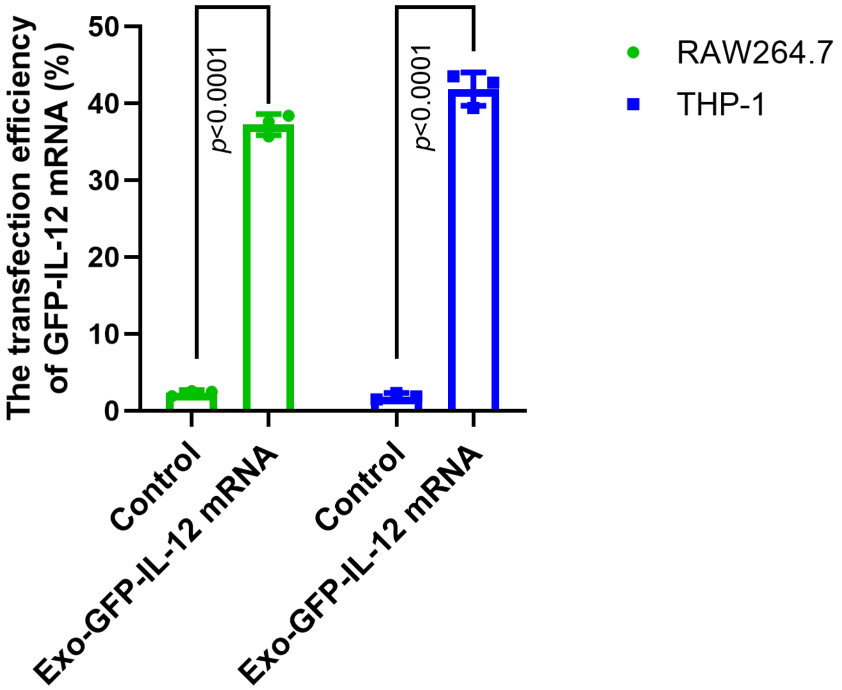

Figure S7. IL-12 mRNA can be stably expressed in RAW264.7 cells and THP-1 cells. Flow cytometry analysis of RAW264.7 and THP-1 cells following 24 h co-incubation with Exo-GFP-IL-12 mRNA, demonstrating efficient transfection and intracellular expression. Exo-GFP-IL-12 mRNA refers to Exo loaded with GFP-labeled IL-12 mRNA for tracking and quantification. Data are presented as mean ± s.d.; two-sided unpaired Student's t-tests were applied for comparisons.

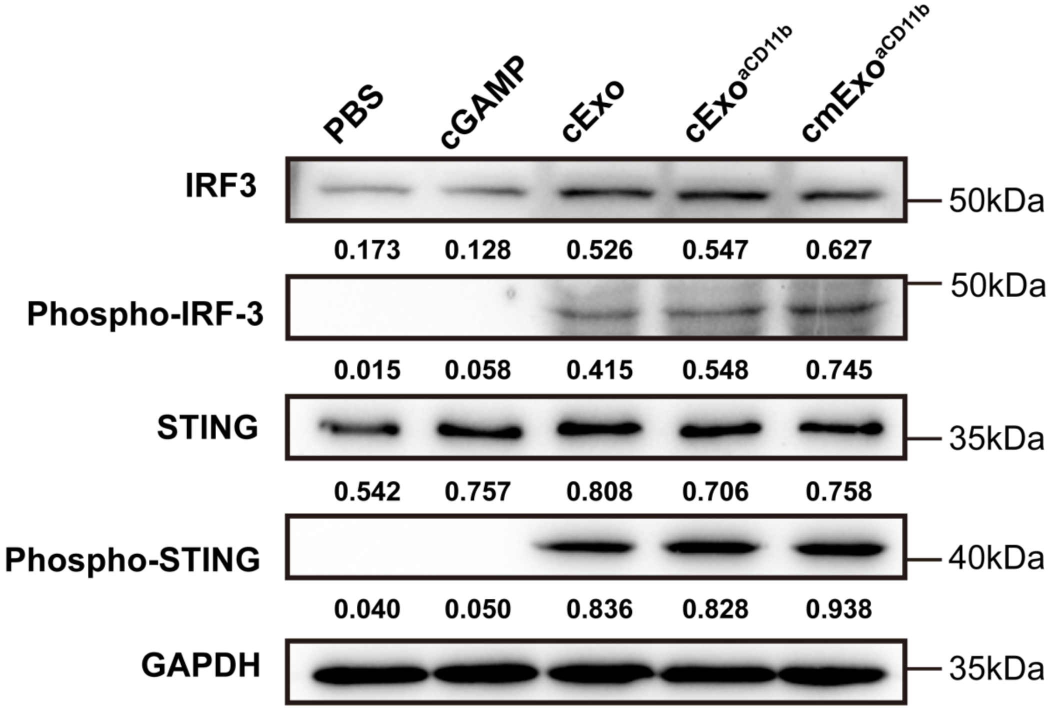

Figure S8. Effects of cmExo^{aCD11b} on STING-IRF3 signaling in THP-1 cells. Western blot analysis of STING-IRF3 pathway proteins in THP-1 cells with different treatments for 6 h. Band intensities were quantified using ImageJ software, and the expression of each protein was normalized to the corresponding GAPDH signal.

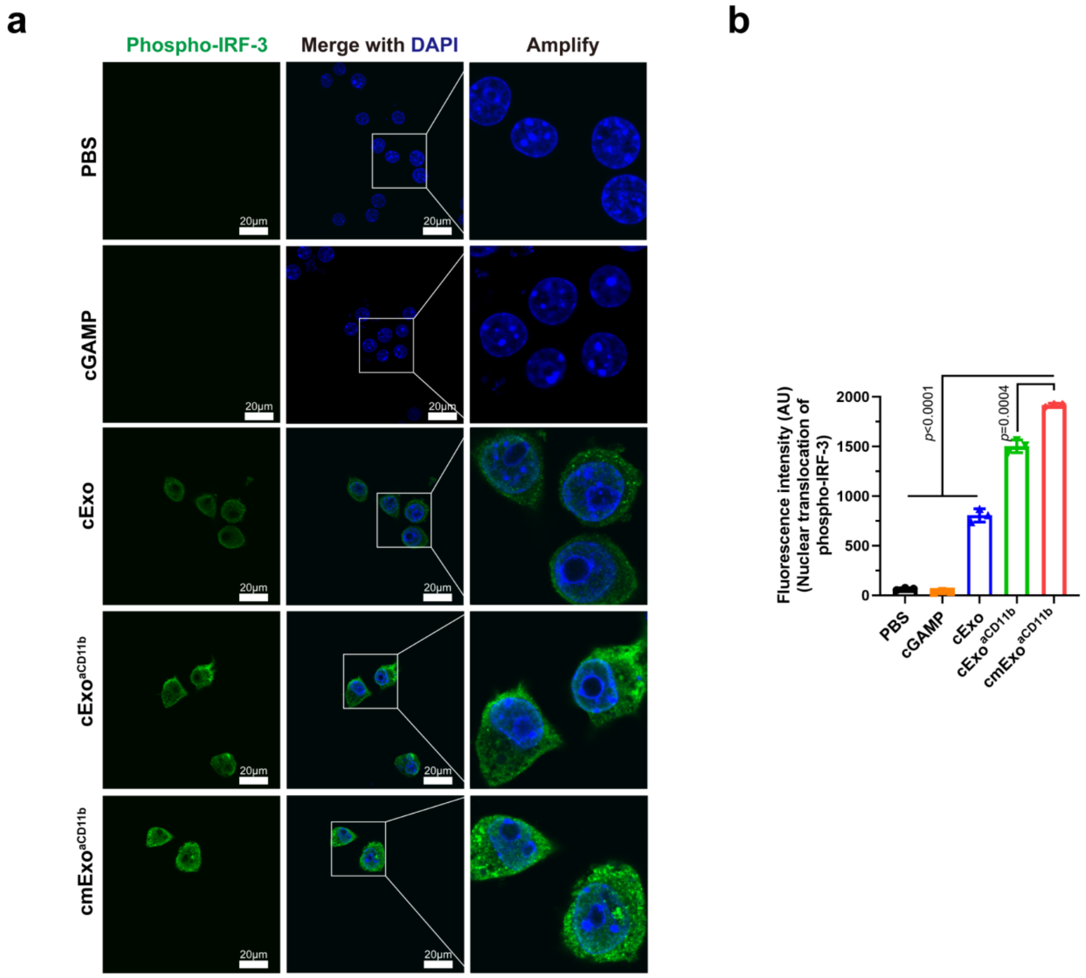

Figure S9. Confocal laser scanning microscopy detects the occurrence of nuclear translocation of phospho-IRF-3 in THP-1 cells after different administration treatments. a. Confocal laser scanning microscopy images of nuclear translocation of activated IRF3 (Phospho-IRF-3, green) in THP-1 cells (blue) at 6 h after cmExo^{aCD11b} and other groups treatment. Scale bar: 20 µm. b. Average fluorescence intensity of phospho-IRF-3 nuclear translocations in each group (n = 3). Data are presented as mean ± s.d.; ~~two-sided unpaired Student's t-tests were applied for comparisons~~.

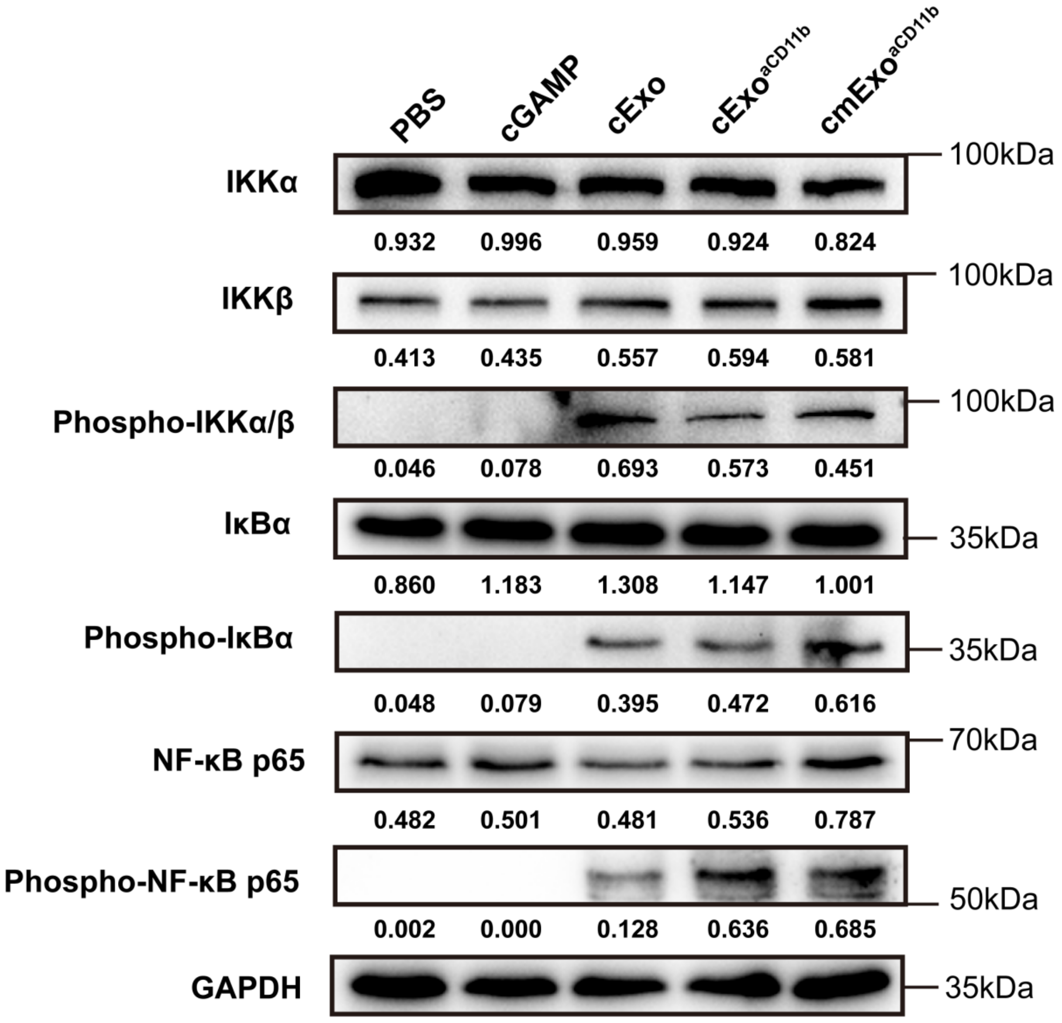

Figure S10. Effects of cmExo^{aCD11b} on the NF-κB p65 pathway in THP-1 cells. Western blot analysis of activated NF-κB p65 in THP-1 cells with different treatments for 6 h. Band intensities were quantified using ImageJ software, and the expression of each protein was normalized to the corresponding GAPDH signal.

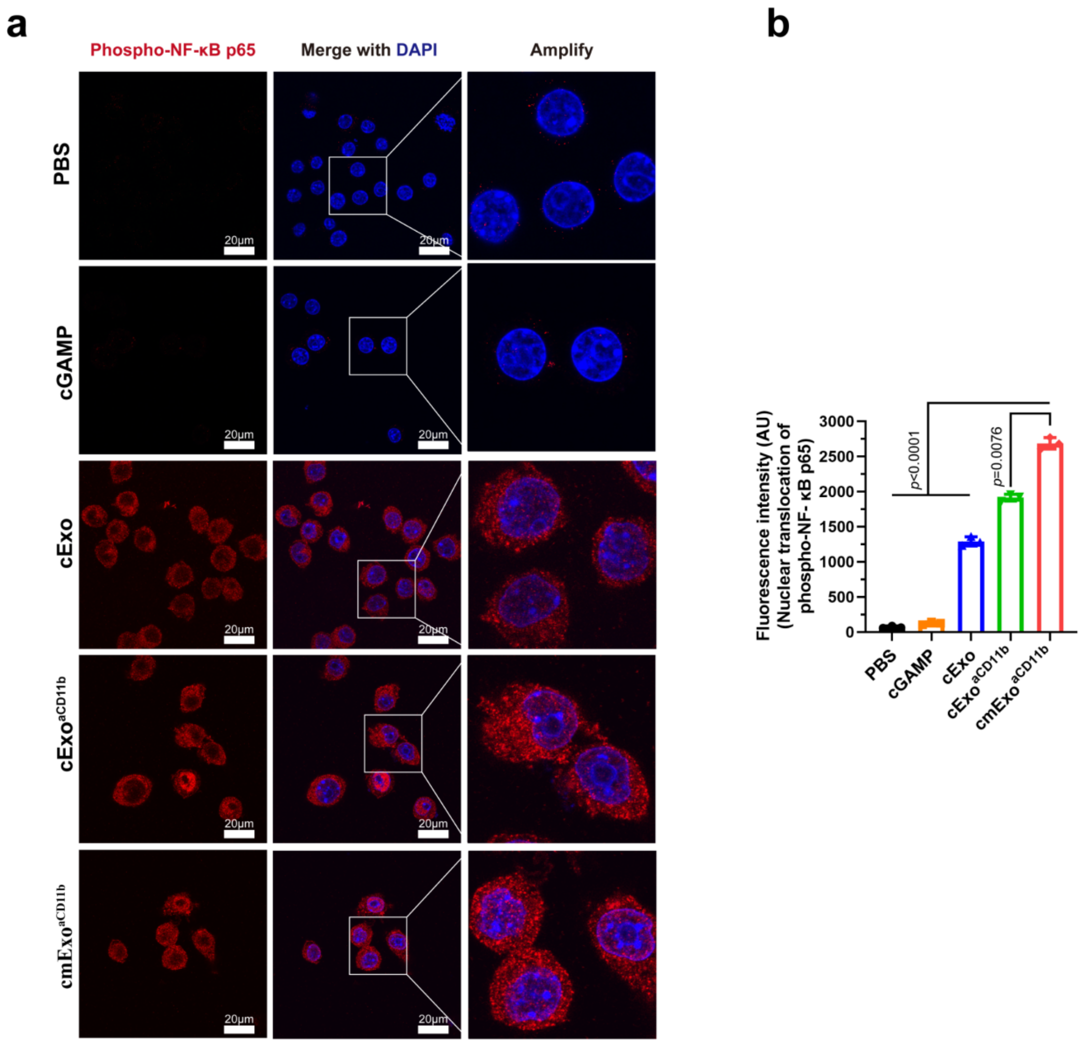

Figure S11. Confocal laser scanning microscopy detects the occurrence of nuclear ectasia of phospho-NF-κB p65 in THP-1 cells after different administration treatments. a. Confocal laser scanning microscopy images of the nuclear translocation of activated NF-κB (p65) in THP-1 cells. Representative immunofluorescence images show that both cExo^{aCD11b} and cmExo^{aCD11b} undergo nuclear translocation, with cmExo^{aCD11b} having a stronger nuclear translocation effect. Scale bar: 20 μm. b. Average fluorescence intensity of NF-κB p65 nuclear translocations in each group (n = 3). Data are presented as mean ± s.d.; ~~two-sided unpaired Student's t-tests were applied for comparisons.~~

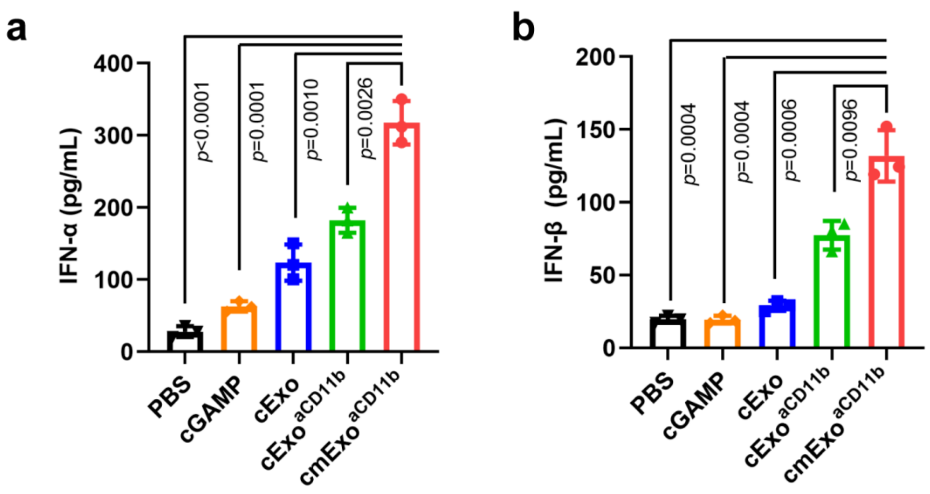

Figure S12. Ability of THP-1 cells to produce IFN-α and IFN-β after each group of treatments. a, b. Levels of IFN-α, IFN-β in THP-1 cell supernatants, as detected by ELISA (n = 3). Data are presented as mean ± s.d.; ~~two-sided unpaired Student's t-tests were applied for comparisons~~.

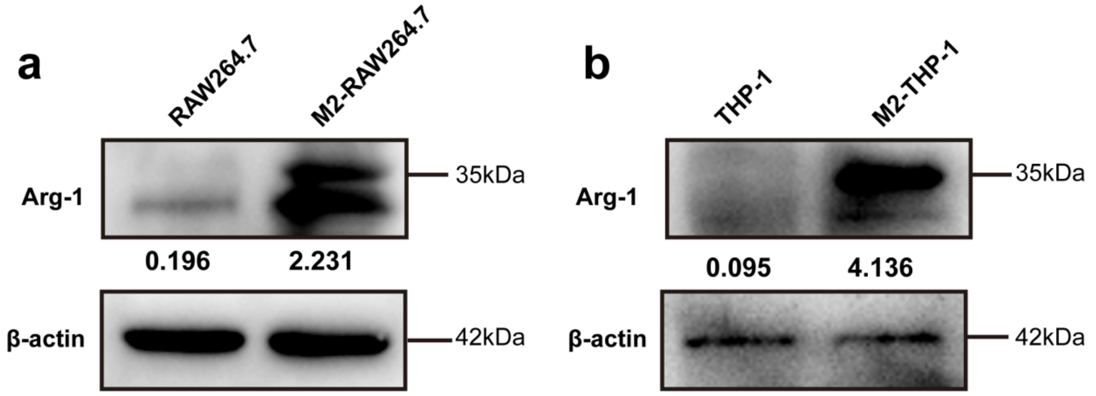

Figure S13. Western blot analysis of Arg-1 protein expression in macrophages after M2 polarization. (a) RAW264.7 cells and (b) THP-1 cells exhibited increased expression of arginase-1 (Arg-1) after M2 polarization, confirming successful induction of the M2 macrophage phenotype. Band intensities were quantified using ImageJ software, with Arg-1 levels normalized to GAPDH as a loading control.

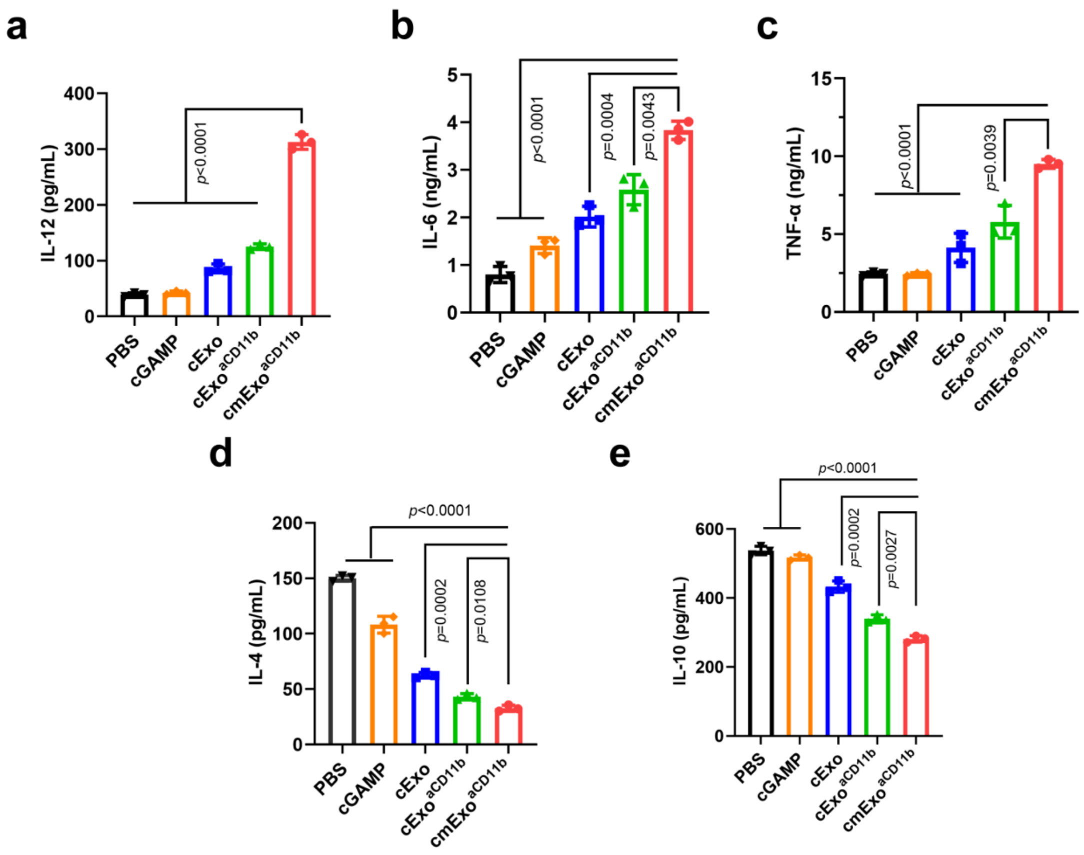

Figure S14. Release of inflammatory cytokines from THP-1 cells after the administration of treatment in each group. a-e. Levels of IL-12, IL-6, TNF-α, IL-4 and IL-10 in THP-1 cell supernatants, as detected by ELISA (n = 3). Data are presented as mean ± s.d.; ~~two-sided unpaired Student's t-tests were applied for comparisons~~.

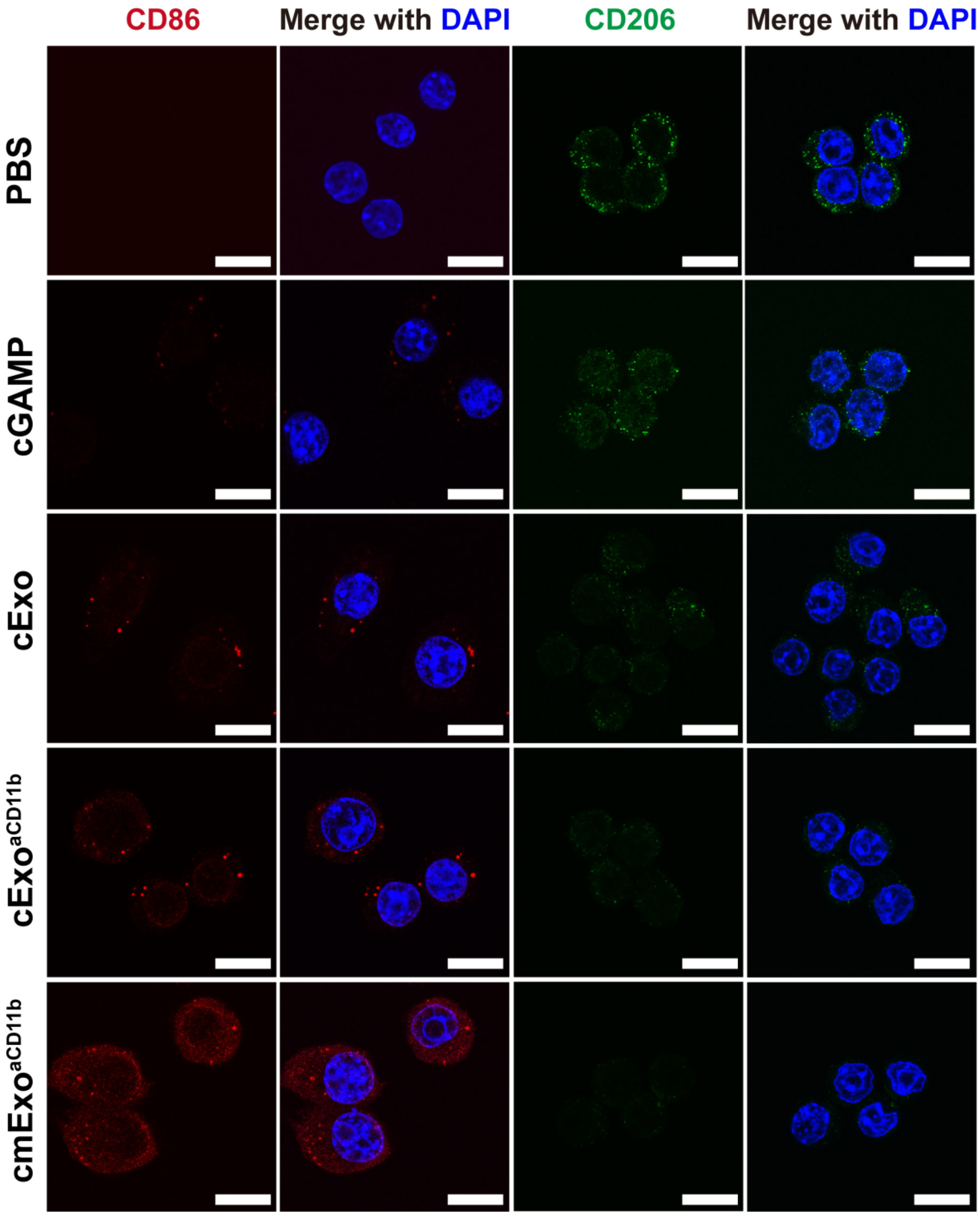

Figure S15. Changes in cell surface CD86 and CD206 expression of THP-1 cells after each group of administration treatment. Expression of CD86 and CD206 in THP-1 cells, as detected by confocal laser scanning microscopy (n = 3). Scale bar: 10 μm.

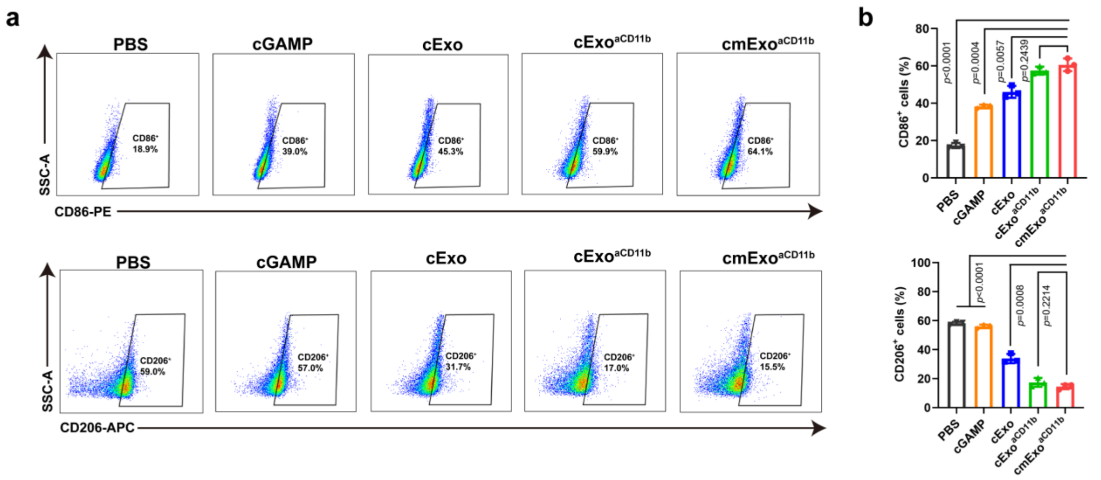

Figure S16. Changes in cell surface CD86 and CD206 expression of THP-1 cells after each group of administration treatment. a. Expression of CD86 and CD206 in THP-1 cells, as detected by flow cytometry (n = 3). b. Proliferation ratio of $CD86^+$ and $CD206^+$ cells in macrophages (n = 3). Data are presented as mean ± s.d.; ~~two-sided unpaired Student's t-tests were applied for comparisons.~~

a

b

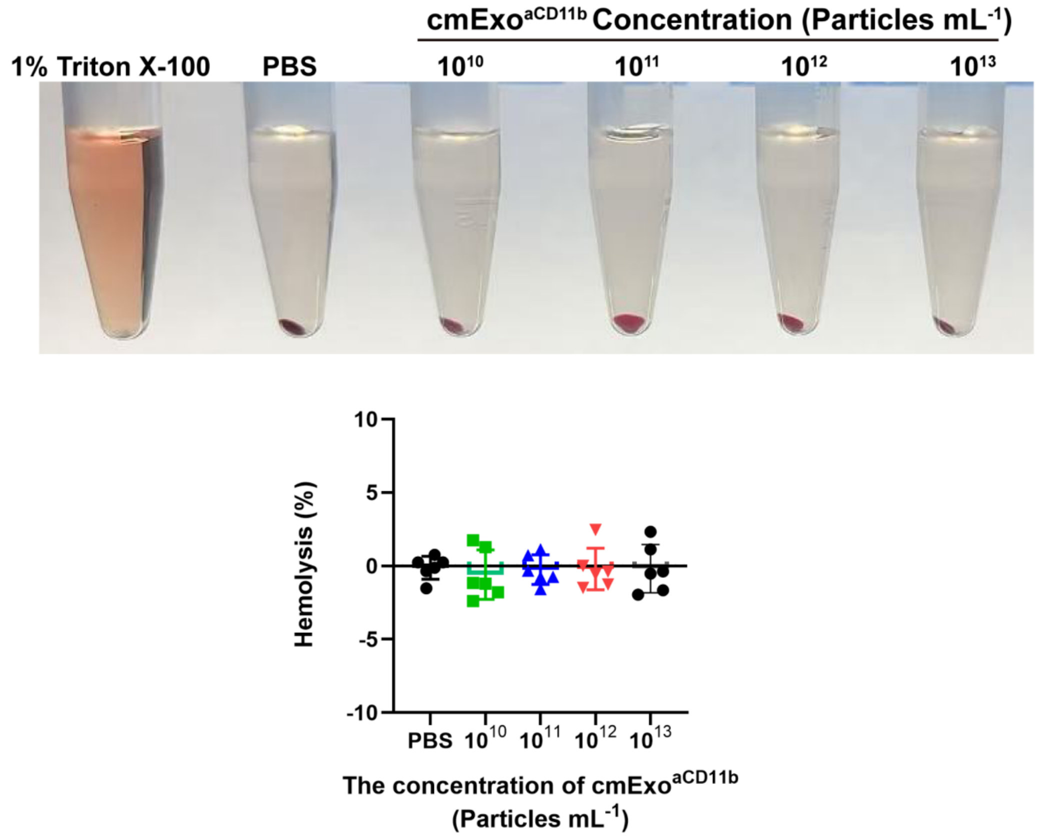

Figure S17. Biosafety of cmExo^{aCD11b}. a. Hemolysis of red blood cells treated with cmExo^{aCD11b}. b. Hemolysis rate measured in each group (n = 6).

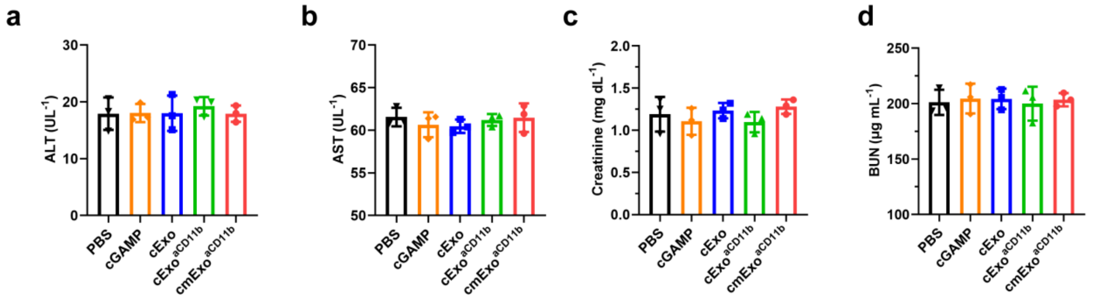

Figure S18. Effects of the administered treatments on the biochemical indices in the blood of mice in each group. a-d. Biochemical indexes including ALT, AST, creatinine, and BUN detected in each group (n = 3).

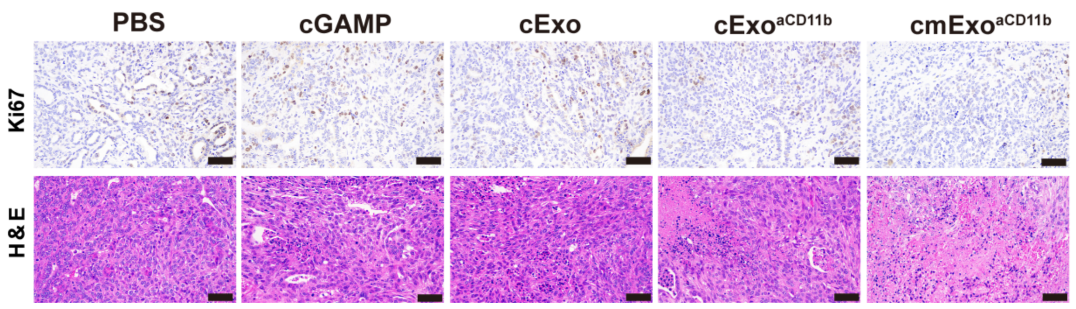

Figure S19. Analysis of Ki67 and H&E staining of tumor tissues. Ki67 and H&E staining of tumor tissues showing excellent therapeutic effect of cmExo^{aCD11b} (n = 3). Scale bar: 100 μm.

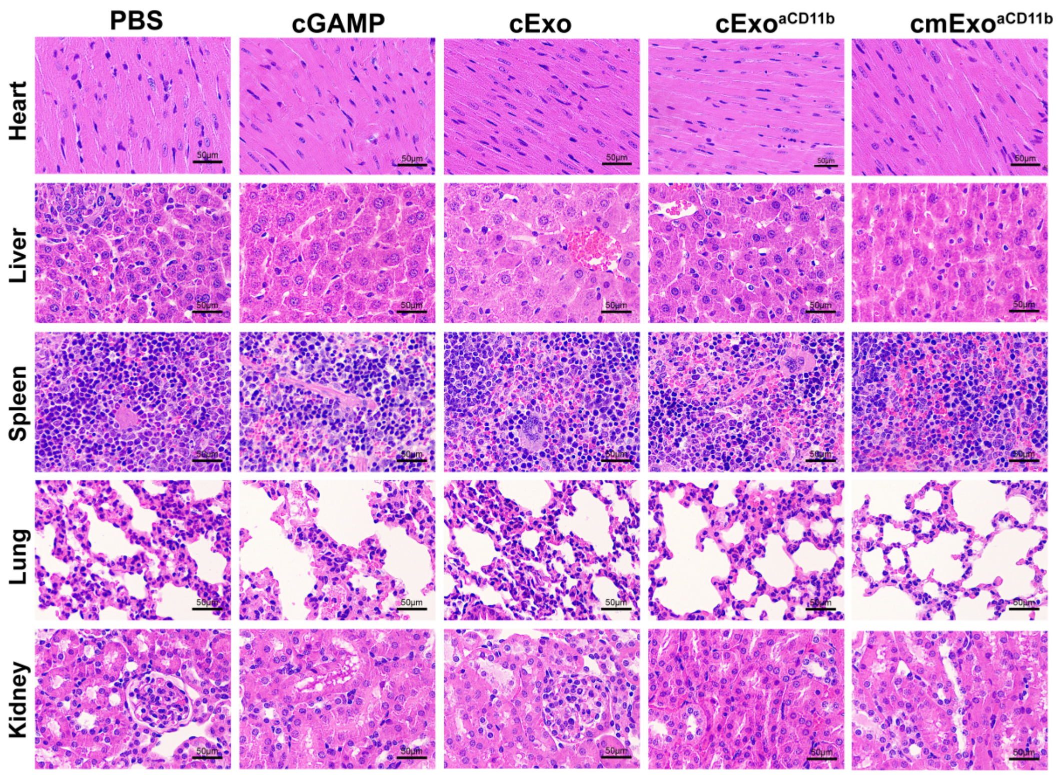

Figure S20. Biosafety of each group of treatments. H&E staining of major organs (heart, liver, spleen, lung, and kidney) of mice (n = 3). No significant histopathological abnormalities were observed, indicating that cmExo^{aCD11b} treatment does not induce overt organ toxicity. Scale bar: 50 μm.

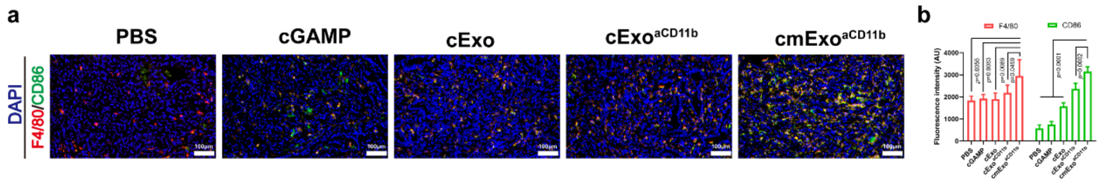

Figure S21. Immunofluorescence staining analysis of M1 macrophage surface markers F4/80 and CD86 on tumors. a. Immunofluorescence staining of CD86 and F4/80 in tumor tissues. Scale bar: 100 μm. b. Quantification of average fluorescence intensity of F4/80 and CD86 protein. Data are presented as mean ± s.d.; ~~two-sided unpaired Student's t-tests were applied for comparisons~~.

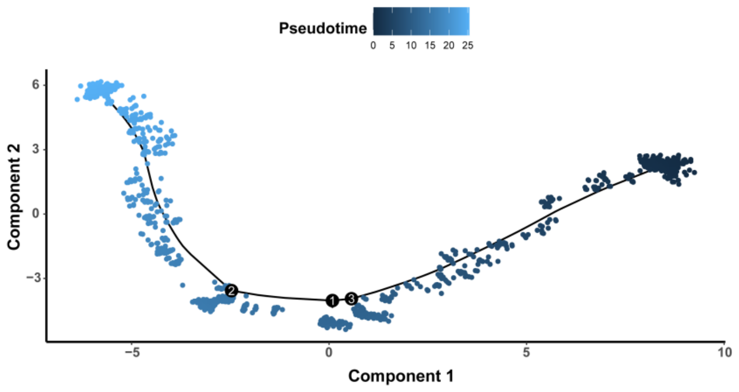

Figure S22. Monocle pseudotime trajectory analysis of macrophage population.

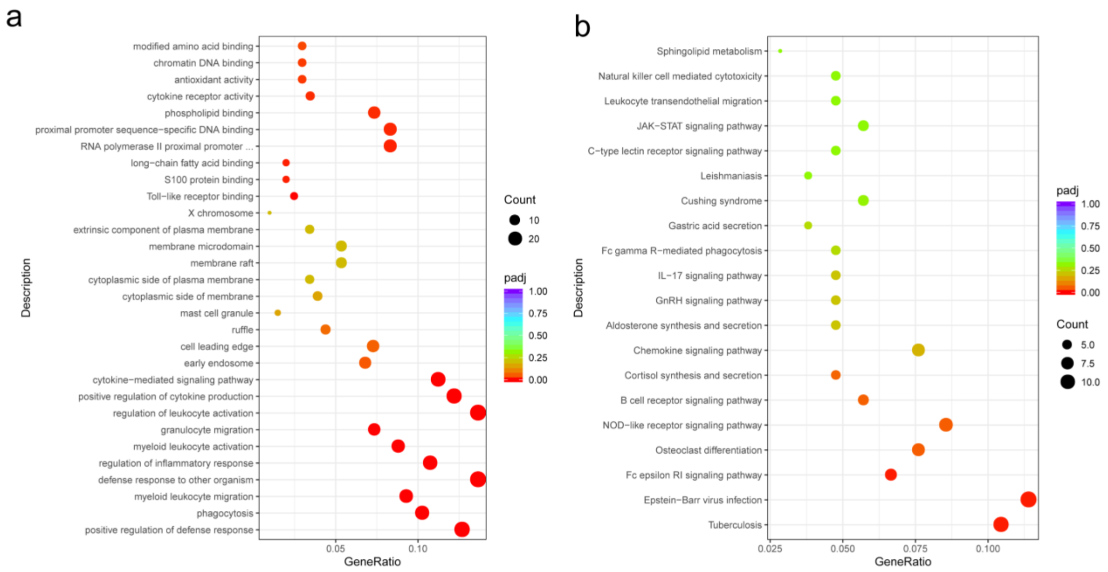

Figure S23. Functional enrichment analyses during macrophage repolarization. a. Bubble plots depicting the progression of gene upregulation during M2-to-M1 repolarization in the GO enrichment analysis. b. Bubble plots illustrating the progression of gene upregulation during M2-to-M1 repolarization in the KEGG pathway analysis.

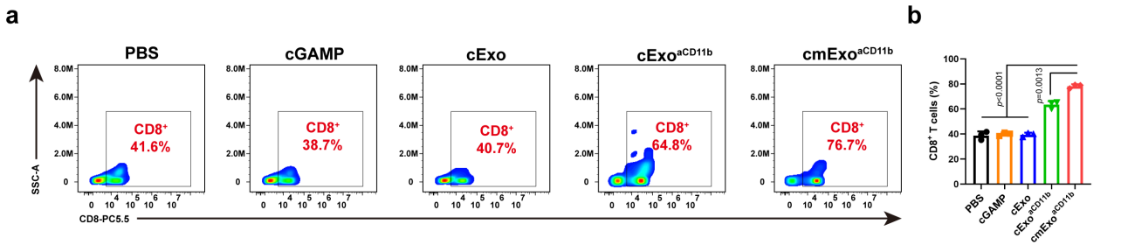

Figure S24. Changes in $CD8^+$ T cell populations in the spleens of tumor-bearing mice. a. Flow cytometry analysis of $CD8^+$ T cells in spleens across treatment groups. b. Quantification of $CD8^+$ T cell proportions in each group (n = 3). Data are presented as mean ± s.d.; two-sided unpaired Student's t-tests were applied for comparisons.

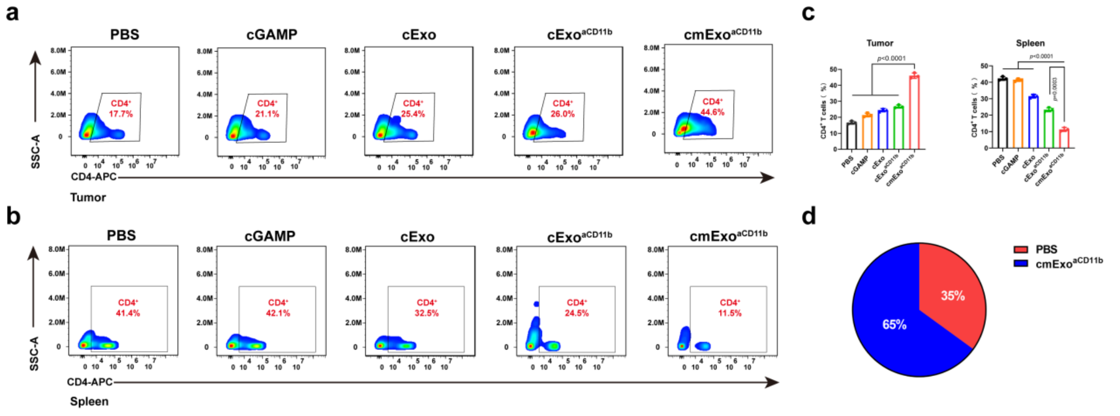

Figure S25. Alterations in CD4$^+$ T cell populations in tumor and spleen by flow cytometry and scRNA-seq. a, b. Flow cytometry analysis of CD4$^+$ T cell proportions in tumors and spleens of mice across treatment groups (n = 3). c. Representative plots showing the percentage of CD4$^+$ T cells in each group. d. Quantification of CD4$^+$ T cells in tumor tissues based on scRNA-seq under different treatment conditions. Data are presented as mean ± s.d.; ~~two-sided unpaired Student's t-tests were applied for comparisons.~~

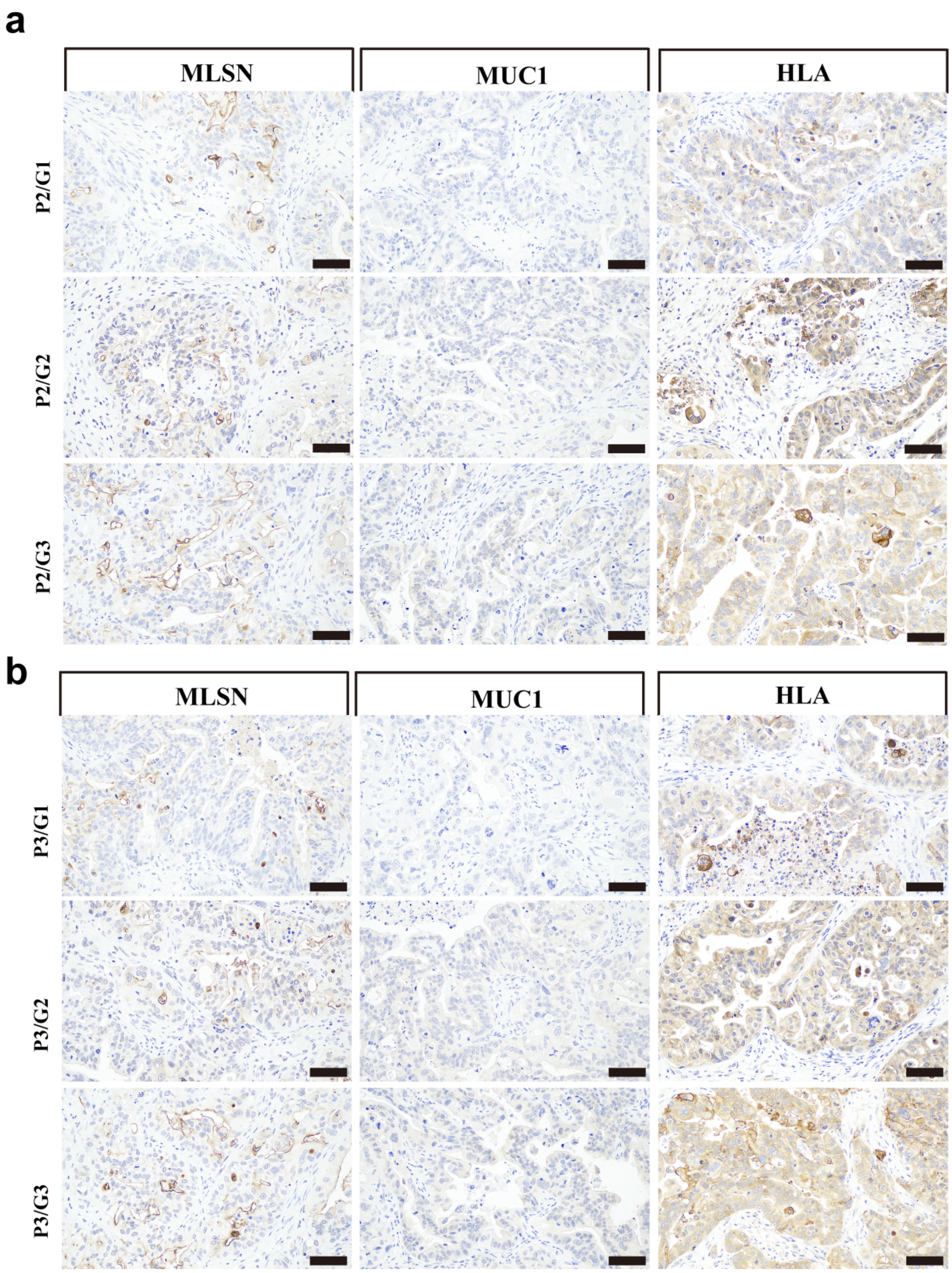

Figure S26. Immunohistochemical characterization of pancreatic cancer tissue from two additional patients across serial passages. a. Immunohistochemical staining for MLSN, MUC1, and HLA in tumor tissues derived from Patient 2 (P2) across three successive passages. Scale bar: 100 μm. b. Immunohistochemical staining for MLSN, MUC1, and HLA in tumor tissues derived from Patient 3 (P3) across three successive passages. Scale bar: 100 μm.

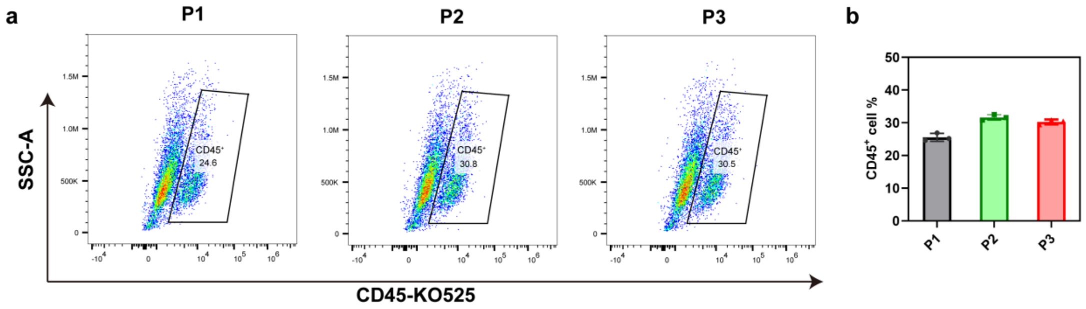

Figure S27. Immune cell engraftment in the PDX mouse model. a, b. Flow cytometry analysis of $CD45^{+}$ cells in PBMCs from PDX mice. Administration of $cmExo^{aCD11b}$ was initiated once the proportion of $CD45^{+}$ cells reached 30%, indicating successful immune cell reconstitution *in vivo*.

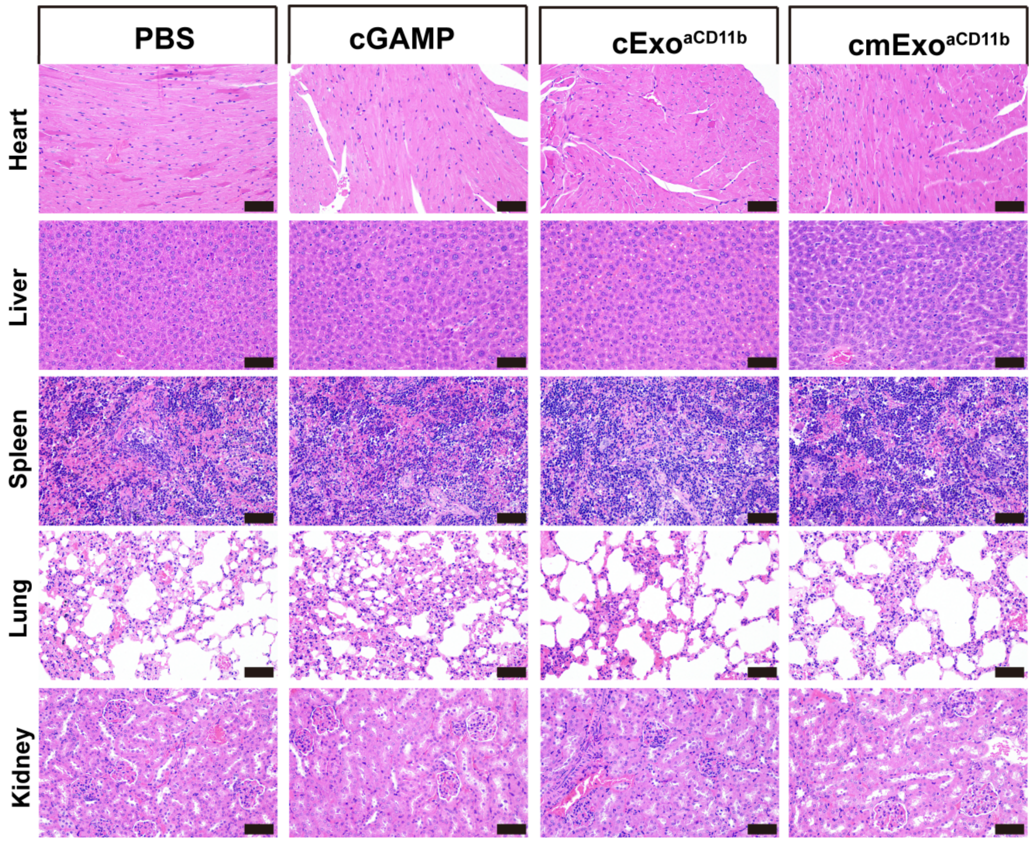

Figure S28. Evaluation of systemic toxicity following treatment. H&E staining of major organs (heart, liver, spleen, lung, and kidney) from PDX mice in each treatment group (n = 3). No observable histopathological abnormalities were found, indicating the favorable biosafety profile of cmExo^{aCD11b}. Scale bar: 100 μm.

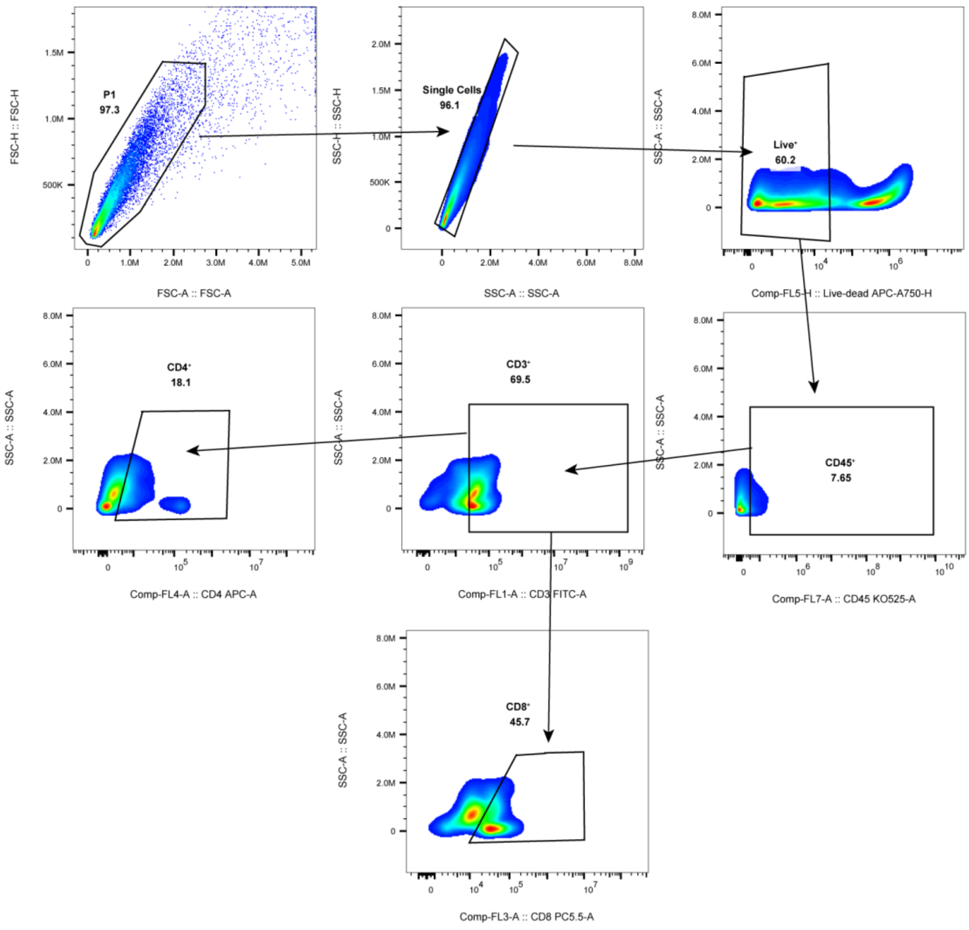

Figure S29. Representative flow cytometry T cell gating strategies.

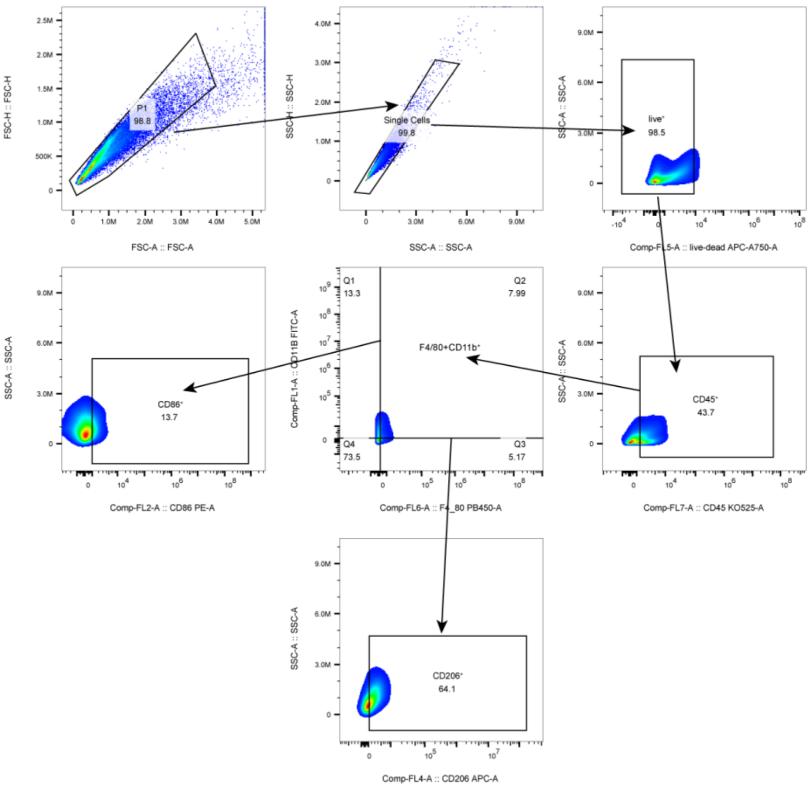

Figure S30. **Representative flow cytometry macrophage gating strategies.**
